# Time matters: genetic composition and evaluation of effective population size in temperate coastal fish species

**DOI:** 10.7717/peerj.9098

**Published:** 2020-05-01

**Authors:** Sara M. Francisco, Joana I. Robalo

**Affiliations:** MARE—Marine and Environmental Sciences Centre, ISPA—Instituto Universitário, Lisbon, Portugal

**Keywords:** Effective population size, mtDNA, nDNA, Temporal structure, Temporal stability, Temporal method

## Abstract

**Background:**

Extensive knowledge on the genetic characterization of marine organisms has been assembled, mainly concerning the spatial distribution and structuring of populations. Temporal monitoring assesses not only the stability in genetic composition but also its trajectory over time, providing critical information for the accurate forecast of changes in genetic diversity of marine populations, particularly important for both fisheries and endangered species management. We assessed fluctuations in genetic composition among different sampling periods in the western Portuguese shore in three fish species.

**Methods:**

White seabream *Diplodus sargus*, sand smelt *Atherina presbyter* and shanny *Lipophrys pholis* were chosen, because of their genetic patterns in distinct ecological environments, insight into historical and contemporary factors influencing population effective size (*N*_*e*_), and degree of commercial exploitation. Samples were obtained near Lisbon between 2003 and 2014 and screened for genetic variation with mitochondrial and nuclear markers. Analyses included genealogies, genetic diversities, temporal structures and contemporary *N*_*e*_.

**Results:**

For mtDNA no temporal structure was detected, while for nDNA significant differences were recorded between some sampling periods for the shanny and the sand smelt. Haplotype networks revealed deep genealogies, with various levels of diversification. The shanny revealed a smaller *N*_*e*_/generation when compared to the other species, which, in turn, revealed no evidence of genetic drift for most study periods. These results highlight the fact that temporal variations in genetic pool composition should be considered when evaluating the population structure of fish species with long distance dispersal, which are more vulnerable to recruitment fluctuations.

## Introduction

Over the last four decades, we have assembled extensive knowledge on the genetic characterization of marine organisms, mainly concerning the spatial distribution and structuring of populations (e.g., Knutsen et al., 2007; Plank et al., 2010; Robalo et al., 2013; Riginos et al., 2019; Verry et al., 2020). With this information, it is now possible and relevant to understand how these patterns behave through time. Temporal monitoring assesses the genetic composition’s stability and trajectories over time, providing critical information for the accurate forecast of changes in the genetic diversity of marine populations. This is increasingly important for both endangered and commercially exploited species, particularly in a context of severe broad-based anthropogenic influences and climate change (Schwartz, Luikart & Waples, 2007; Cuveliers et al., 2011; Goldstien et al., 2013).

Temporally replicated sampling also allows the estimate of *N*_*e*_—effective population size, one of the most important parameters in both conservation and evolutionary biology (Waples & Do, 2010; Hare et al., 2011). From a genetic perspective, *N*_*e*_ is defined as the size of an ideal population that has the same rate of change in allele frequencies and heterozygosity as the observed population (Wright, 1931). Estimates of *N*_*e*_ may be used to assess the loss of genetic variation, increases in inbreeding, accumulation of mutations, and effectiveness of selection (Waples & Do, 2010; Hare et al., 2011). In the short to medium term, potential reductions in *N*_*e*_ may lead to inbreeding depression and/or drastic loss of genetic diversity (Frankham, Bradshaw & Brook, 2014), particularly in commercially overexploited populations, populations inhabiting highly degraded ecosystems or located near the margins of the species distribution. Loss of genetic diversity may take a long time to recover through mutation, thus limiting or impeding the adaptive response to environmental changes (Lynch & Lande, 1998).

Several approaches have been developed in the past decades to overcome difficulties in directly measuring *N*_*e*_ in the marine realm (e.g., Wang, 2005; Luikart et al., 2010; Hare et al., 2011; Wang, Santiago & Caballero, 2016 for a review). Traditional demographic estimators based on Capture-Mark-Recapture (e.g., Jolly-Seber Model (Jolly, 1965; Seber, 1965), and multinomial approach in open populations (Crosbie & Manly, 1985)) have been complemented with genetic methods: single-sample *N*_*e*_ estimators (e.g., linkage disequilibrium method (Hill, 1981), heterozygote-excess method (Pudovkin, Zaykin & Hedgecock, 1996), sibship frequency (Wang, 2009), molecular coancestry (Nomura, 2008)) and two-sample *N*_*e*_ estimators (temporal change in allele frequencies (Waples, 1989; Wang, 2001)).

A frequently used genetic approach to measure contemporary changes in *N*_*e*_ has been the Temporal Method (Nei & Tajima, 1981). This method depends on estimates of allelic frequencies in two or more points in time, assuming that the observed genetic drift between two sampling moments will be more pronounced in small populations. Jorde & Ryman (1995) adjusted the method to incorporate the effects of age structure, demonstrating that the magnitude of changes in allelic frequencies was also determined by age-specific birth and survival rates, in addition to the actual size and the sampling interval. Later, these authors presented a direct extension of the previous model, allowing its application to temporal samplings of single cohorts born within a given number of years (Jorde & Ryman, 2007; Jorde, 2012).

Temporal genetic changes in coastal species have been linked to the stochastic nature of oceanographic conditions, including upwelling systems, fronts, eddies and sharp discontinuities in physicochemical variables (Selkoe et al., 2006; White et al., 2010). Moreover, the stability of allele frequencies in natural populations can be deeply influenced by extreme events (anthropogenic and/or natural disturbing factors) (Allison et al., 2003). Additionally, intrinsic biological and ecological factors associated with the species life cycle, also shape the genetic pattern of marine organisms, namely inshore-offshore spawning, pelagic larval duration and other early life-history traits (Riginos & Victor, 2001; Galarza et al., 2009).

Genetic monitoring through time, specifically, the temporal method, has been successfully employed to evaluate the temporal stability and estimate *N*_*e*_ in several species, including some stocks with high economic significance such as the European hake *Merluccius merluccius* (Pita et al., 2017), the brown trout *Salmo trutta* (Serbezov et al., 2012) and the Atlantic cod *Gadus morhua* (Therkildsen et al., 2010). In the present study, three coastal fishes were chosen to cover a wide range of contrasting environmental and ecological traits: the white seabream *Diplodus sargus* L. 1758 (Pisces: Sparidae); the sand-smelt *Atherina presbyter* Cuvier 1829 (Pisces: Atherinidae); and the shanny *Lipophrys pholis* L. 1758 (Pisces: Blenniidae).

Although they are not threatened—the three species are listed as least concerned—their population trend is unknown, and they can be locally vulnerable due to various reasons. The white-seabream is a commercially important species throughout European shores, wide-spread and locally abundant (Pollard et al., 2014) and, although no major threats have been identified, some local overfishing may occur and lead to reduction in population size. The sand-smelt is commercially exploited as life bait for tuna and local coastal development can be a threat (Gon, 2015). In the long-term, climate change could be a problem for this temperate species. Finally, no major threats are found across the shanny’s distribution range (Williams & Craig, 2014). This species can be used as an indicator for pollution monitoring due to several of its characteristics, including restricted home range and high sensitiveness to organic contaminants (Lima et al., 2008).

These target species provide crucial insight into historical and present factors influencing effective population sizes and genetic patterns in distinct ecological environments and degrees of commercial interest. In this study, we assessed the changes in genetic composition in different points in time in the western Portuguese shore, using a mitochondrial and a nuclear marker (chosen due to their extensive use in the past decade). The specific objectives were: (1) to compare the inter-annual variation in genetic composition and structure of coastal species with contrasting traits, and (2) to test the temporal model and its potential for *N*_*e*_ estimation in populations with overlapping generations.

## Materials & Methods

### Target species

The white seabream *Diplodus sargus* is a coastal species in the north-eastern Atlantic ranging from Senegal to the Bay of Biscay, including the archipelagos of Canaries, Madeira and Azores, Mediterranean and Black Sea (Bauchot & Hureau, 1986). Eggs are planktonic, hatching after 3 days at 15–17 °C (Morato et al., 2003). Larvae are also planktonic, settling after a pelagic larval duration (PLD) of 14–19 days (Di Franco et al., 2011). Adults have considerable swimming ability and tend to remain near the coast. This is a commercially exploited fish, with increasing landings per year in the last 40 years (FAO, 2019). Some degree of overfishing can be empirically inferred, even if stocks show no evidence of decline, since larger *D. sargus* are mainly spotted within Marine Protected Areas. Previous studies on the genetic structure of the white seabream revealed isolation-by-distance (IBD), suggesting genetic isolation over large geographic distances (e.g., Domingues et al., 2007). In contrast, allozyme studies reported significant divergences in cohorts sampled over a 6-month period in a Mediterranean population (Lenfant & Planes, 2002; Planes & Lenfant, 2002).

The sand smelt *Atherina presbyter* is an inshore marine species, also present in estuaries and coastal lagoons. Its distribution area comprises the British Isles and southern North Sea to the Canary Islands and Mauritania (Quignard & Pras, 1986), and has also been reported in the Azores (Santos, Porteiro & Barreiros, 1997). This species spawns in shallow waters, the eggs are demersal and attached to vegetation, and larvae hatch after 15–16 days with 6.7–7.5 mm TL at a temperature of 15 °C (Bamber, Henderson & Turnpenny, 1985). The larval stage is very short (the hatching larvae are well developed and ready to start exogenous feeding), which likely restricts passive dispersal. Migratory movements of adult sand-smelts along exposed shores are probably difficult, although they are active swimmers in the water column. Our current understanding of gene flow in *A. presbyter* indicates a structured population across its range, also showing IBD (Francisco et al., 2009). A previous mtDNA study revealed temporal stability in the genetic composition of the sand-smelt from the Portuguese west coast (Francisco & Robalo, 2015).

The shanny *Lipophrys pholis* is a rocky intertidal resident fish, very common in western European shores, ranging from Norway to Mauritania and from the Azores and Madeira to the entrance of the Mediterranean (Zander, 1986). The eggs are demersal, guarded by the male, and hatch after 16 days with 5.0 mm total length at a temperature of 17 °C (Almada et al., 1992; Faria et al., 2002). The larvae hatch in a well-developed stage and settle at 13–14 mm TL after a PLD of ca. 29 days at a temperature of 15.5–17.5 °C (Faria et al., 2002). Juveniles and adults show weak swimming capabilities and, consequently, restricted movements within the same rocky stretch (Faria, Almada & Goncalves, 1996). Previous molecular work on the shanny, using both nuclear and mitochondrial markers, strongly suggests panmixia across its distribution (Francisco, Vieira & Almada, 2006; Stefanni et al., 2006; Francisco et al., 2011). Contrary to *A. presbyter*, the mtDNA study of *L. pholis* detected significant genetic differentiation between some sampling years (Francisco & Robalo, 2015).

### Sampling scheme

Individuals of *L. pholis*, *D. sargus* and *A. presbyter* were sampled near Lisbon, in S. Pedro do Estoril (38°42′N, 9°22′W) and Fonte-da-Telha (38°34′N, 9°11′W) (Table 1) between 2003 and 2014. Both sampling locations are in the vicinity of marine protected areas and present heterogeneous rocky habitats mixed with sandy patches (e.g., Henriques, Gonçalves & Almada, 1999; Faria & Almada, 2001). They are known to harbour a relevant number of post-larvae and juveniles for several taxa, being important settlement and recruitment areas (e.g., Borges et al., 2009; Vinagre et al., 2018). Juveniles of each year were collected in intertidal rocky pools (*L. pholis*) and beach channels (*D. sargus* and *A. presbyter*). A small sample of fin clip was collected, preserved in 96° ethanol and deposited in ISPA-IU/MARE collections. All sampling and handling of fish were conducted according to established animal welfare guidelines (ORBEA-ISPA, Animal Welfare Body) and following the relevant legislation, as none of the sampled species are endangered or protected in Portugal.

**Table 1:** Diversity measures for sampling periods of *Diplodus sargus*, *Atherina presbyter* and *Lipophrys pholis* based on the control region of the mitochondria: number of sequences (*N*), number of haplotypes (*Nh*), percentage of private haplotypes (%Ph), haplotype richness (*R*), private allelic richness (*pR*), haplotype diversity (*h*), nucleotide diversity (*π*) and mean number of pairwise differences (*k*).

Species	Sampling period	*N*	*N*_*h*_	*%Ph*	*R*	*pR*	h	*π*	*k*
*Diplodus sargus*	2006	20	19	68.42	20.000	16.008	0.995	0.034	13.058
	2009	39	30	70.00	16.441	11.55	0.974	0.032	12.093
	2011	27	22	59.09	15.379	9.476	0.983	0.033	12.479
	2014	95	73	79.45	16.431	11.643	0.993	0.029	11.135
	All	181	120	–	–	–	0.990	0.031	11.690
*Atherina presbyter*	2005	34	29	68.96	20.235	12.423	0.986	0.022	8.012
	2012	91	63	69.84	17.640	9.352	0.984	0.021	7.780
	2013	61	48	68.08	17.973	9.396	0.992	0.021	7.866
	2014	95	56	62.50	16.541	7.922	0.975	0.022	8.054
	All	281	155	–	–	–	0.984	0.021	7.931
*Lipophrys pholis*	2003	30	26	69.23	21.634	13.211	0.991	0.031	11.614
	2013	97	73	87.61	23.708	15.291	0.988	0.028	10.844
	2014	99	88	93.18	26.240	18.034	0.997	0.031	11.977
	All	226	171	–	–	–	0.995	0.030	11.522

### DNA extraction, amplification and sequencing

Total genomic DNA was extracted from about 20 mg of tissue with REDEXtract-N-mp kit (Sigma-Aldrich) following manufacturer’s instructions. Polymerase Chain Reaction (PCR) amplification was performed for two fragments, the mitochondrial control region (CR) and an intron of the nuclear S7 ribosomal protein gene (S7), with the primer pairs: LPro1 (5′-ACT CTC ACC CCT AGC TCC CAA AG-3′) and HDL1 (5′-CCT GAA GTA GGA ACC AGA TGC CAG-3′) (CR for the three species) (Ostellari et al., 1996), S7RPEX1F (5′-TGG CCT CTT CCT TGG CCG TC-3′) and S7RPEX2R (5′-AAC TCG TCT GGC TTT TCG CC-3′) (first intron of the S7 for the shanny and the white seabream), S7RPEX2F (5′-AGC GCC AAA ATA GTG AAG CC-3′) and S7RPEX3R (5′-GCC TTC AGG TCA GAG TTC AT-3′) (second intron of the S7 for the sand-smelt) (Chow & Hazama, 1998). The PCR protocol was performed in a 20 µl total reaction volume with 10 µl of REDExtract-N-ampl PCR mix (Sigma-Aldrich), 0.8 µl of each primer (10 µM), 4.4 µl of Sigma water and 4 µl of template DNA. An initial denaturation at 94 °C for 3 min was followed by 35 cycles (denaturation at 94 °C for 30/45 s, annealing at 55/58 °C for 30/45 s, and extension at 72 °C for 1 min; values CR/S7, respectively) and a final extension at 72 °C for 10 min on a Bio-Rad MyCycler thermal cycler. The same primers were used for the sequencing reaction and PCR products were purified and sequenced at STABVIDA (Portugal, http://www.stabvida.net) and GATC (Germany, http://www.gatc-biotech.com).

Sequences were edited with Codon Code Aligner (http://www.codoncode.com/index.htm) and aligned with Clustal X2 (Larkin et al., 2007). For S7 both strands of the same specimen were recovered, whenever possible, following the approach of Sousa-Santos et al. (2005). This approach takes advantage of the presence of indels in each nuclear marker and uses them to accurately reconstruct the individual haplotypes without the need for probabilistic estimation. Sequences were deposited in GenBank (Accession numbers MG992598 –MG992888, MH024090 –MH024357, MH030878 –MH031272). Additional sequences from previous work (Francisco et al., 2006; Francisco, Vieira & Almada, 2006; Domingues et al., 2007; Francisco et al., 2008; Francisco & Robalo, 2015) were retrieved from GenBank (Tables S1–S3 in Data S1).

### DNA analyses

The appropriate model of sequence evolution for the CR and S7 of each species was determined using the software jModeltest v.2.1.10 (Guindon & Gascuel, 2003; Darriba et al., 2012), under the Akaike Information Criterion (AIC) (Nei & Kumar, 2000). Parsimony networks estimated with TCS version 1.21 (Clement, Posada & Crandall, 2000) were used to analyse relationships among haplotypes and to compute outgroup weights, based on parsimony methods (Templeton, Crandall & Sing, 1992). Visualization and network layout were edited with tcsBU (Múrias dos Santos et al., 2016).

ARLEQUIN software package v.3.5 (Excoffier & Lischer, 2010) was used to estimate the genetic diversity (*k*—mean number of pairwise differences (Tajima, 1983); *π*- nucleotide diversity; and *h*—haplotype diversity (Nei, 1987)) within each sampling period, to perform analyses of molecular variance (AMOVA) (Excoffier, Smouse & Quattro, 1992) and to compute pairwise *FST*s. The *χ*2 test (Salicru et al., 1993) was used to access the significance of differences in haplotype diversity among temporal samples. The software HP-Rare (Kalinowski, 2005) was used to estimate allelic richness *R* and private allelic richness *pR*, using rarefaction to correct for sample-size bias. Principal Coordinate Analysis (PCoA) was performed with GenAlEx 6.5 (Peakall & Smouse, 2006; Peakall & Smouse, 2012) to visualize the patterns of temporal genetic structure in a bi-dimensional space. The analyses of the S7 intron were also run in ARLEQUIN, after allowing the program to reconstruct the haplotypes present, using the ELB algorithm (Excoffier, Laval & Balding, 2003). The same software was used to perform the exact probability tests for deviations from the Hardy–Weinberg equilibrium (HWE) (Guo & Thompson, 1992).

Contemporary effective population size (*N*_*e*_) and genetic drift (*Fs*) were estimated using TempoFs (Jorde & Ryman, 2007), under the temporal method of allele frequency shifts. The program reports *Fs*′ (genetic drift corrected for sampling plan) and *Ne* per generation. For this approach, we used sampling plan II (individuals sampled before reproduction and not returned to the population; Waples, 1989) and a generation time of 2 yr for *L. pholis* (Milton, 1983; Faria, Almada & Goncalves, 1996), 2 yr for *D. sargus* (Morato et al., 2003) and 1 yr for *A. presbyter* (Turnpenny, Bamber & Henderson, 1981).

## Results

### Mitochondrial data

For *D. sargus*, a total of 181 CR sequences were obtained, comprising 120 haplotypes and 119 polymorphic sites (117 transitions, 15 transversions and two indels) (Table S1). For the seabream CRs, 16.67% of haplotypes were shared between sampling periods. For *A. presbyter*, 281 CR sequences were obtained corresponding to 155 haplotypes (368bp long fragment). Differences among haplotypes corresponded to 70 polymorphic sites (63 transitions, 19 transversions and six indels) (Table S2). For the sand-smelt, 16.13% of haplotypes were shared between sampling periods. A total of 226 shannies were sequenced for the CR (380 bp), corresponding to 171 distinct haplotypes (Table S3). A total of 108 polymorphic sites were found, corresponding to 105 transitions, 26 transversions and eight indels. Only 7.02% of haplotypes were shared among sampling periods. The generalized time-reversible (GTR) + invariable sites (I) (Tavare, 1986) was estimated as the optimal molecular evolutionary model for the CR of the three species.

**Figure 1:**
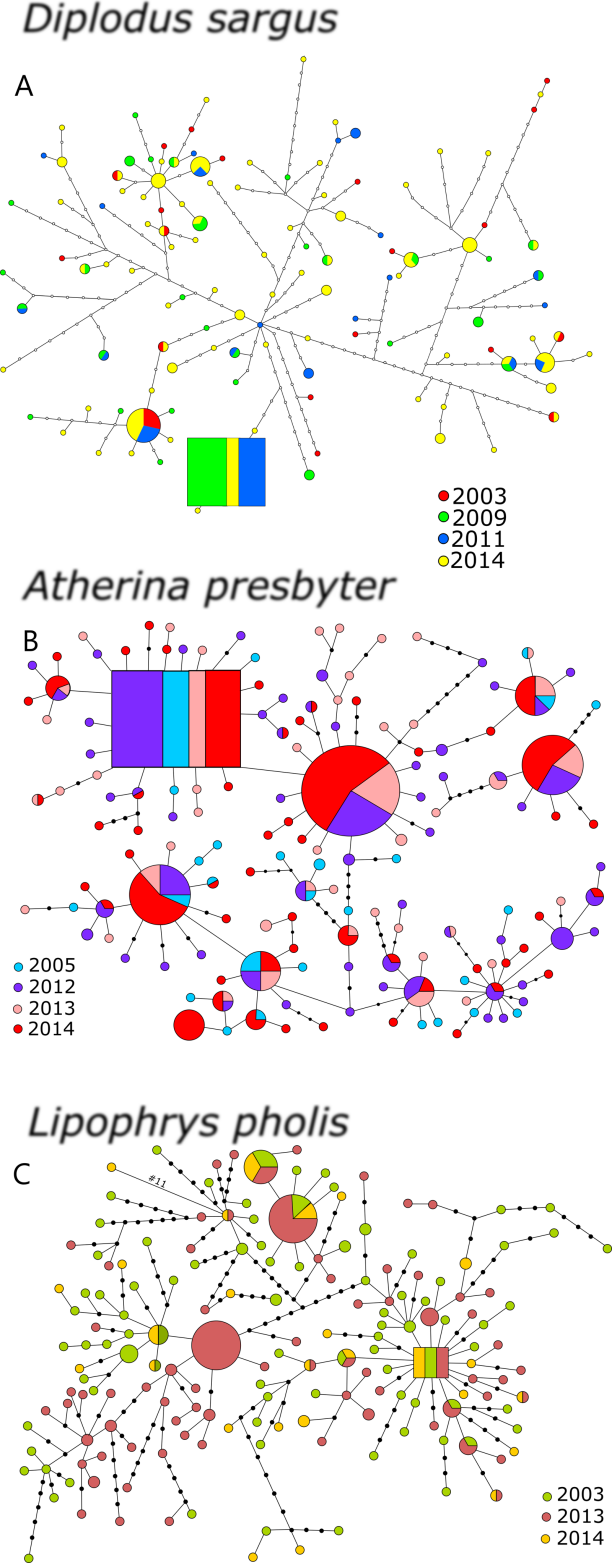
Haplotype network for the CR of (A) *Diplodus sargus*, (B) *Atherina presbyter* and (C) *Lipophrys pholis*. The haplotype with the highest out group probability is displayed as a square, other haplotypes as circles. The area of the circles is proportional to each haplotype frequency. Colours refer to the year of sampling. In the case where haplotypes are shared among sampling periods, shading is proportional to the frequency of the haplotype in each period.

The statistical parsimony networks constructed with the CR datasets revealed multiple levels of diversification and deep genealogies, seemingly without temporal structure (Fig. 1). The inferred ancestral haplotype for the CR of *D. sargus* included specimens collected in 2009, 2011 and 2014 (outgroup weight: 0.071). In this network, most of the haplotypes were not arranged in a star-like pattern, and some of the branches reached a maximum of 46 mutational steps, and 18 steps from the ancestral haplotype. For *A. presbyter*, the network built with the CR dataset presented several star-like patterns around haplotypes, including the haplotype inferred as ancestral (outgroup weight: 0.051), which comprised individuals from every sampling period. The network was dominated by two haplotypes shared by most sampling periods. In this network, also deep but with less diversification, some of the haplotypes differed 16 mutational steps from ancestor. For *L. pholis*, the CR network displayed several haplotypes, including the estimated ancestor, in the centre of star-like patterns. This ancestral haplotype included specimens collected in the three sampling periods (outgroup weight: 0.053), and some haplotypes differed as many as 17 mutations (Fig. 1). Interestingly, for all three species, haplotypes that initially had been merely inferred were sampled in more recent periods.

For the CR, genetic diversity indices were generally high and similar across years (Table 1). Salicru tests revealed non-significantly different levels of haplotype diversity among sampling years: *χ*^2^ = 1.557 (*p* = 0.816), *χ*^2^ = 4.342 (*p* = 0.362) and *χ*^2^ = 3.022 (*p* = 0.388), for *D. sargus*, *A. presbyter* and *L. pholis* respectively. The CR allelic richness and private allelic richness yielded distinct results for the three species, with no pattern for the seabream, increasing over sampling time in the shanny, and decreasing in the sand-smelt (Table 1).

Global genetic differentiation among sampling years was not significant for CR in any of the analysed species—AMOVA results (white seabream: *FST* = 0.003, *p* = 0.250; sand-smelt: *FST* = 0.002, *p* = 0.312; shanny: *FST* = 0.009, *p* = 0.074). Comparisons between periods were not significant in *D. sargus* and *A. presbyter*. However, for *L. pholis*, significant differentiation was detected between 2013 and 2014 samples (*FST* = 0.013, *p* = 0.028). No species showed marked patterns of temporal genetic structure in CR and, therefore, the PCoA was not performed.

Estimates of effective population size based on the CR of the three species are given on Table 2, after correction for haploid data. *L. pholis* revealed a smaller *N*_*e*_/generation when compared to the other species and *A. presbyter* showed no evidence of genetic drift for some of the study periods (negative values of *Fs*′), which resulted in much higher *N*_*e*_/generation (∼inf).

**Table 2:** Estimates of contemporary effective population size and genetic drift for *Diplodus sargus*, *Atherina presbyter* and *Lipophrys pholis*. Mitochondrial control region (CR), nuclear S7 ribosomal protein gene (S7), estimated drift (*Fs*′), estimated effective population size per generation (*N*_*e*_∕*gen*).

Species	Marker	Sampling interval	*Fs*′	*N*_*e*_∕*gen*
*Diplodus sargus*	CR	2006–2009	0.029	102
		2009–2011	−0.005	∞
		2011–2014	0.005	426
	S7	2006–2009	0.022	68
		2009–2011	−0.008	∞
		2011–2014	−0.005	∞
*Atherina presbyter*	CR	2005–2012	−0.001	∞
		2012–2013	−0.001	∞
		2013–2014	0.001	1,282
	S7	2005–2012	0.352	10
		2012–2013	0.015	34
		2013–2014	−0.006	∞
*Lipophrys pholis*	CR	2003–2013	0.008	612
		2013–2014	0.010	94
	S7	2003–2013	0.534	5
		2013–2014	0.010	53

### Nuclear data

For *D. sargus,* 302 sequences of S7 were obtained with 308bp (corresponding to 151 individuals), comprising 99 haplotypes and 50 polymorphic sites (29 transitions, 21 transversions and 2 indels) (Table S1). Only 12.12% of haplotypes of S7 were shared between sampling periods for the seabream. The amplification of S7 (*N* = 286,572 sequences) in *A. presbyter* resulted in a 201bp fragment, corresponding to 35 haplotypes (Table S2). A total of 25 polymorphic sites (6 transitions, 12 transversions and 7 indels) were found, and 57.14% of the haplotypes were shared between sampling periods. For the S7 of the shanny, a total of 360 sequences (180 individuals) were obtained (576 bp), comprising 24 distinct haplotypes (26.09% shared between sampling periods) (Table S3). The fragment yielded 20 polymorphic sites, corresponding to 9 transitions, 8 transversions and 3 indels. The unequal transitional substitution (TIM3) + invariable sites (I) + rate variation among sites (G) (Posada, 2003) was estimated as the optimal molecular evolutionary model for the S7 of the seabream. For the S7 of the sand-smelt, the selected model was the equal transitional substitution model (TIM1ef) + invariable sites (I) (Posada, 2003), and for the shanny was the unequal transitional substitution model (TIM3) + invariable sites (I) (Posada, 2003).

**Figure 2:**
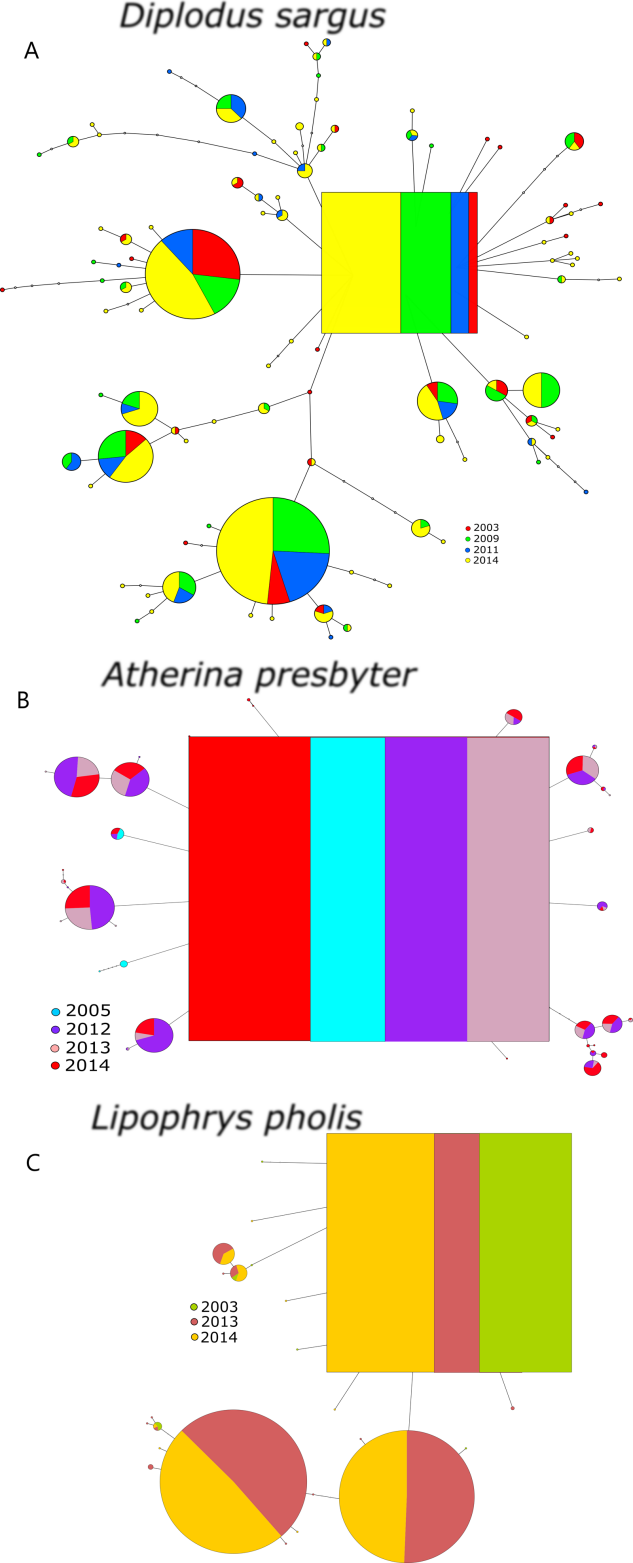
Haplotype network for the S7 of (A) *Diplodus sargus*, (B) *Atherina presbyter* and (C) *Lipophrys pholis*. The haplotype with the highest outgroup probability is displayed as a square, other haplotypes as circles. The area of the circles is proportional to each haplotype frequency. Colours refer to the year of sampling. In the case where haplotypes are shared among sampling periods, shading is proportional to the frequency of the haplotype in each period.

The S7 haplotype networks of the three species revealed shallower genealogies comparing to the CR (Fig. 2). The networks showed star-like patterns dominated by 2–3 very frequent haplotypes and no temporal structure was evident. The inferred ancestral haplotypes were shared among sampling periods, yielding outgroup weights of 0.067, 0.117 and 0.190, for *D. sargus*, *A. presbyter* and *L. pholis*, respectively.

The S7 genetic diversity indices of the three species are summarized in Table 3. The pattern of haplotypic diversity stability across years registered for the CR was only recovered for the S7 of *D. sargus* (*χ*^2^ = 1.289, *p* = 0.863). Salicru tests yielded significant differences for the haplotype diversity between sampling year for both *A. presbyter* (*χ*^2^ = 73.080, *p* < 0.001) and *L. pholis* (*χ*^2^ = 17.864, *p* < 0.001). Similarly, the Markov chain tests yielded significant deviations from the HWE for some years of the sand-smelt and shanny datasets (*p* < 0.001) (Table 3).

**Table 3:** Diversity measures for sampling periods of *Diplodus sargus*, *Atherina presbyter* and *Lipophrys pholis* based on the S7: number of sequences (*N*), number of haplotypes (*Nh*), percentage of private haplotypes (%Ph), haplotype richness (*R*), private allelic richness (*pR*), haplotype diversity (*h*), nucleotide diversity (*π*), mean number of pairwise differences (*k*) and test of deviations from the Hardy–Weinberg equilibrium observed/expected heterozygosity (*Ho/He*).

Species	Sampling period	*N*	*N*_*h*_	*%Ph*	*R*	*pR*	h	*π*	*k*	*Ho/He*
*Diplodus sargus*	2006	20	20	90.00	24.961	15.461	0.963	0.012	3.709	1.000/0.963
	2009	35	33	69.70	20.583	8.081	0.942	0.013	3.968	0.943/0.942
	2011	19	19	78.95	19.000	8.052	0.953	0.012	3.863	0.895/0.953
	2014	77	67	86.57	24.368	11.301	0.961	0.013	4.006	0.961/0.962
	All	151	125	–	–	–	0.981	0.013	3.948	–
*Atherina presbyter*	2005	38	4	50.00	4.000	2.070	0.242	0.002	0.380	0.034/0.033 ^*^
	2012	90	17	17.65	14.334	2.082	0.816	0.001	1.990	0.129/0.142^*^
	2013	64	19	21.05	16.223	4.262	0.743	0.009	1.772	0.099/0.099
	2014	94	26	26.92	17.830	4.605	0.770	0.010	2.063	0.102/0.115
	All	286	42	–	–	–	0.734	0.009	1.779	–
*Lipophrys pholis*	2003	20	7	51.71	7.000	4.637	0.360	0.002	1.121	0.400/0.360
	2013	76	13	53.85	7.162	2.608	0.757	0.004	2.534	0.750/0.757^*^
	2014	84	12	50.00	6.225	1.489	0.721	0.004	2.327	0.655/0.721^*^
	All	180	23	–	–	–	0.734	0.004	2.380	–

**Notes.**

* Significant values of probability *p*.

**Figure 3:**
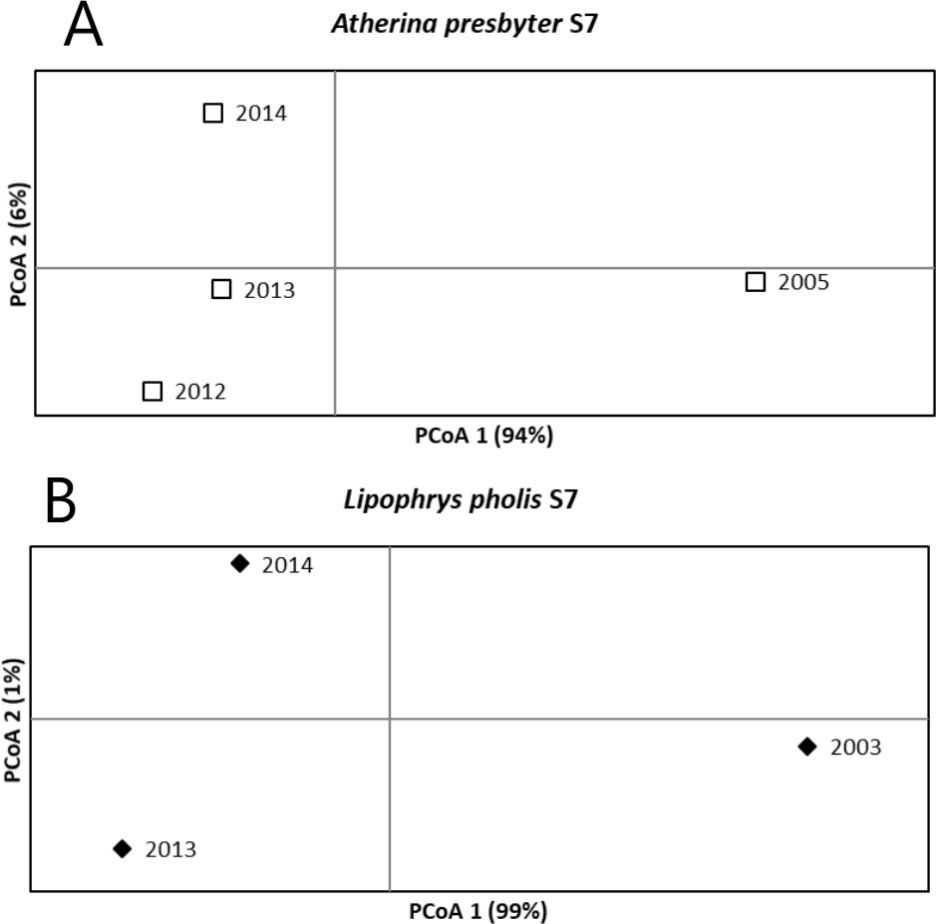
Principal Coordinate Analysis for the S7 of (A) *Atherina presbyter* and (B) *Lipophrys pholis*.

For the S7 of the white seabream, the AMOVA results revealed no significant temporal structure among sampling years (*FST* = 0.003, *p* = 0.204). Pairwise *FST* comparisons were also non-significant. A different pattern was recovered for the S7 of the sand-smelt and the shanny, as significant global genetic differentiation was yielded (*FST* = 0.020 *p* < 0.001 and *FST* = 0.067 *p* < 0.001, respectively). For *A. presbyter* significant differences were found between 2005 and every other period (*p* < 0.001). For *L. pholis* pairwise *FST* was significant between 2003 and the more recent sampling times (*p* < 0.001). The PCoA corroborated these patterns of temporal genetic structure, with the first axis explaining 94% and 99% of the observed variance, for the S7 of *A. presbyter* and *L. pholis*, respectively, and suggesting two groups associated with older vs recent sampling periods (Fig. 3).

For the S7 fragment, TempoFs yielded lower *N*_*e*_/generation estimates for the shanny (Table 2). Both the white seabream and the sand-smelt revealed negative values of *Fs*′ for some of the more recent sampling intervals, i.e., no evidence of genetic drift and, conversely, very high *N*_*e*_/generation values (∼inf) (Table 2).

## Discussion

### Temporal genetic stability

Traditionally population genetic and phylogeographic studies have disregarded the temporal dimension, pooling samples based on their geographical origin and ignoring their collecting period (Viñas, Alvarado-Bremer & Pla, 2004; Charrier et al., 2006; Luttikhuizen et al., 2008; Gonzalez-Wanguemert et al., 2011; Stefanni et al., 2015). One of our main result is the subsequent appearance of inferred haplotypes, clearly reinforcing the importance of temporal evaluations. Similar results were also found using mitochondrial markers in several other species (e.g., *Genypterus capensis* (Henriques et al., 2017), *Prionace glauca* (Veríssimo et al., 2017), *Seriola lalandi* in the Pacific Ocean (Sepúlveda & González, 2017)).

Another important finding was the stability across years in these three ecologically distinct species, reinforcing the preliminary work by Francisco & Robalo (2015). This high degree of similarity was found in (a) the high genetic diversity, with little variation between sampling periods, especially true for the mitochondrial data (Table 1); (b) the deep genealogies with several diversification levels (Fig. 2); and (c) the absence of temporal structure for the mitochondrial data. In contrast, we detected temporal structure and some marked differences among sampling years in both the shanny and the sand-smelt for S7 (Fig. 3). In fact, this differentiation between sampling periods was also detected for the CR of *L. pholis*, notwithstanding the absence of temporal genetic structure.

Stochasticity in larval survival and transport, due to variation in oceanographic conditions, and genetic differentiation among temporal samples are broadly documented in the literature (e.g., Selkoe et al., 2006; Selkoe et al., 2010). *Lipohrys pholis* lays fewer eggs and has a longer planktonic larval stage when compared to both *A. presbyter* and *D. sargus*. Therefore, while its male-guarding behaviour can promote larval retention near rocky coasts, this is easily disrupted by atypical or severe events, when larvae may be transported very large distances if storm conditions prevail. Thus, the shanny’s higher differentiation (marked differences among sampling periods in both markers and temporal structure for S7) might be closely related with its greater vulnerability to recruitment fluctuations. Conversely, the white seabream revealed a greater degree of temporal stability for both molecular markers. In contrast, allozyme studies in *D. sargus* in the Mediterranean found rapid genetic change within a population, assumed to be driven by genetic drift (Lenfant & Planes, 2002; Planes & Lenfant, 2002). We suggest that these apparently contradictory findings may be due to the type of molecular marker. Planes & Lenfant (2002) associated the large variation in the white seabream’s reproductive success with linkage disequilibrium and genetic relatedness shown in their allozyme data. In our study (mtDNA and nDNA) the absence of temporal structure is probably associated with *D. sargus*’ higher fecundity (Goncalves & Erzini, 2000; Mouine et al., 2007), longer spawning season (Dias et al., 2016) and relatively short PLD (Di Franco et al., 2011). Temporal stability has been described in several other marine species, such as *Solea solea* (Cuveliers et al., 2011), *Pagrus auratus* (Bernal-Ramírez et al., 2003), *Meganyctiphanes norvegica* (Papetti et al., 2005) and *Ammodytes marinus* (Jiménez-Mena et al., 2019).

Also noteworthy is the significant deviation from HWE in the sand-smelt and the shanny, the two species with most contrasting early life-history traits. Although unexpected for a relatively long PLD species like *L. pholis*, this result was previously reported in other marine organisms (e.g., Chapman, Ball & Mash, 2002; Karlsson & Mork, 2005; Pérez-Portela et al., 2019). Departure from HWE has been ascribed to natural selection, migration, Wahlund effect, null alleles, inbreeding and/or phenotypic assortative mating (e.g., Addison & Hart, 2005; Karlsson & Mork, 2005; Gonzalez, Beerli & Zardoya, 2008; Garnier-Géré & Chikhi, 2013). Although we were not able to explain these results, we are aware that HW disequilibrium violates the assumptions behind temporal *N*_*e*_ estimation (e.g., Nei & Tajima, 1981; Serbezov et al., 2012; Wang, Santiago & Caballero, 2016).

### Estimates of contemporary effective population size

In the present work, the estimated population effective size per generation was smaller for the shanny than the other two species, corroborating preliminary findings by Francisco & Robalo (2015). The negative *Fs*′ found for some of the more recent sampling intervals, for both white seabream and sand-smelt, evidenced no genetic drift (CR and S7). Conversely, the *N*_*e*_/generation values for these species were very high (∼inf) (Table 2). Genetic stochasticity is most likely weaker in marine organisms with such large Ne (Palstra & Ruzzante, 2008), as found in fish species like *S. solea* (Cuveliers et al., 2011) and *G. morhua* (Therkildsen et al., 2010) (see Marandel et al. (2019) for a review). Lower *N*_*e*_/generation was found in fish species such as the herring *Clupea harengus* (Larsson et al., 2010), the silver seabream *P. auratus* (Hauser et al., 2002) and the thornback ray *Raja clavata* (Chevolot et al., 2008). The extremely low *N*_*e*_/generation found in the first sampling interval of *L. pholis* and *A. presbyter* (S7) (Table 2) might be an underestimation due to statistical artefacts, immigration from other populations, or other factors (e.g., Palstra & Ruzzante, 2008).

Considering the target species and sampling scheme, the adjusted temporal method was, in our opinion, the best choice for estimating contemporary *N*_*e*_. Not only is this method considered the least biased for species with overlapping generations (e.g., Jorde & Ryman, 2007; Waples & Yokota, 2007), the analysis of consecutive cohorts is also the best way to reduce *N*_*e*_ estimation bias (e.g., Jorde & Ryman, 1995; Luikart et al., 2010). Nevertheless, several factors can confound *N*_*e*_ estimates, including the aforementioned departure from HWE. For instance, temporal estimates of *N*_*e*_ may be biased by potential migratory movements within metapopulations that can override the effects of genetic drift (e.g., Palstra & Ruzzante, 2008; Waples & Do, 2010; Ryman et al., 2014). In fact, the assumption of complete isolation of the study population is frequently violated, and the resulting bias is generally of unknown magnitude (e.g., Ryman et al., 2014). In this study, this might result in biased *N*_*e*_ estimates for the sand-smelt and white seabream, for which IBD patterns have been recorded (Domingues et al., 2007; Francisco et al., 2009). Sampling strategy is another potential source of bias (e.g., Jorde & Ryman, 2007; Waples & Yokota, 2007; Luikart et al., 2010), including low sample size and sex-biased or age-biased sampling. In the present work, uneven sample size between years (considerably lower N for the first sampling point) likely affected our *N*_*e*_ estimates. Species life-history and reproductive strategy can also hamper interpretation of *N*_*e*_ estimates (e.g., Waples & Yokota, 2007). Sequential hermaphrodites, such like the protandrous *D. sargus* (Bauchot & Hureau, 1986), present sex ratios skewed towards the initial sex (Charnov & Bull, 1989). According to standard fixed-sex theory, this results in reduced *N*_*e*_ (Wright, 1938) due to greater genetic drift (Charlesworth, 2009). The very high *N*_*e*_/generation values obtained for the white seabream seemingly contradict this prediction (Table 2). This expected lower *N*_*e*_ was also challenged in recent studies with protogynous species over historical timescales (Coscia et al., 2016), and in analysis of an eco-evolutionary model with ten hypothetical species (Waples, Mariani & Benvenuto, 2018).

Recently, the reliability of contemporary estimates of *N*_*e*_ has been challenged and questioned (e.g., Wang, Santiago & Caballero, 2016; Marandel et al., 2019). However, these reviews focused on single-point estimation approaches (Linkage-Disequilibrium methods), assuming discrete generations and, implicitly, reduced population sizes. Marandel et al. (2019) found significantly biased estimates in simulations of large populations of fish and therefore a need for extra-large sampling sizes (impracticable in most sampling schemes). Unlike the criticized studies, our work used a temporal method comprising extensions to estimate *N*_*e*_ in age-structured species (Jorde, 2012).

While the present study used only one geographical location and two genetic markers, these apparent limitations allowed a rigorous comparison with data from several species collected in the same area over the past fifteen years and using the same two markers (mitochondrial control region and nuclear S7) (e.g., Domingues et al., 2006; Almada et al., 2012; Robalo et al., 2013; Francisco et al., 2014; Stefanni et al., 2015; Almada et al., 2017; Pappalardo et al., 2017), which facilitated comparison and calibration of genetic diversity results. Thus, it was possible to observe the high genetic diversity pattern found in several coastal fish populations in western Portugal (e.g., *D. vulgaris* (Stefanni et al., 2015) and *Labrus bergylta* (Almada et al., 2017)).

## Conclusions

Genetic monitoring through time seeks to disentangle life-trait patterns affecting marine organisms, while deepening our ability to forecast changes in both genetic composition and diversity. This study assessed fluctuations in genetic composition among different sampling periods in the western Portuguese shore using a comparative approach in three species with distinct ecological and life-history characteristics. No temporal structure was detected for the mitochondrial marker, while for nDNA significant differences were recorded among some sampling periods for the shanny and the sand-smelt. The haplotype networks revealed deep genealogies, and one of our major findings was the repeated appearance of previously inferred haplotypes. The shanny revealed a smaller *N*_*e*_/generation when compared to the other species but revealed no evidence of genetic drift for most study periods. These results clearly underline that temporal variation in gene pool composition should be considered when evaluating population structure of long larval dispersion fish species, which are more vulnerable to recruitment fluctuations. Comparison between the commercially and non-commercially explored species yielded no conclusive results.

Previous authors suggested increasing the number of time-point estimates (e.g., from pre-harvest and postharvest times around an overexploitation event) to circumvent difficulties in short-term prediction of *N*_*e*_ (Pita et al., 2017). Future studies with additional time-points covering a longer time span, additional sampling locations covering the species geographical distribution and additional markers would greatly improve the reliability of the present paper’s results. Available tools include a combination of methods of demographic inference and large genomic datasets generated with RAD-seq (restriction site-associated DNA sequencing) (e.g., Barbato et al., 2015), as recently reported in critically endangered species (e.g., *Carcharias taurus* in Reid-Anderson, Bilgmann & Stow, 2019) and commercially important species (e.g., *Oncorhynchus kisutch* in Barría et al., 2019; *Solea solea* in Le Moan et al., 2019). These genomic approaches, with their high-throughput sequencing methods, will likely improve our understanding of recent population demography (Waples, 2016). Applying and extending this framework to species with distinct features (e.g., life-history traits), conservation status and commercial importance would be of paramount importance to detect global patterns and predict the ability of species to adapt to future changes.

##  Supplemental Information

10.7717/peerj.9098/supp-1 Data S1 Sampling periods, specimens and GenBank accession numbers for CR and S7 of *Diplodus sargus, Atherina presbyter* and *Lipophrys pholis.*

## Additional Information and Declarations

### Field Study Permissions

The following information was supplied relating to field study approvals (i.e., approving body and any reference numbers): The sampling took place in accordance with the relevant legislation, as none of the sampled species is endangered or protected in Portugal.

### DNA Deposition

The following information was supplied regarding the deposition of DNA sequences: Sequences are available in GenBank: MG992598–MG992888, MH024090–MH024357, MH030878–MH031272.

### Data Availability

The following information was supplied regarding data availability: The complete list of specimens, sampling year, haplotype number and GenBank accession number for the mitochondrial control region and nuclear S7 ribosomal protein gene (intron 1 and 2) of the three target species (the white seabream Diplodus sargus, the sand-smelt Atherina presbyter and the shanny Lipophrys pholis) are available in the Supplemental File.
