# Supplementary material for: Time matters: genetic composition and evaluation of effective population size in temperate coastal fish species
Source: PeerJ. 2020 May 1;8:e9098. doi: 10.7717/peerj.9098 (PMC7197400; doi:10.7717/peerj.9098)
Supplement: Data S1 [file peerj-08-9098-s001.docx]

**Supplemental Data**

Table S1 – Sampling periods, specimens and Genebank Accession Numbers for CR and S7 of *Diplodus sargus*.

|  |  |  |  |  |
| --- | --- | --- | --- | --- |
| Haplotype number | Specimen | Sampling year | Accession number | Work |
| 1 | SP100 | 2006 | EF468566 | Domingues *et al*., 2007 |
|  | N240 | 2014 | MG992720 | present work |
| 2 | SP102 | 2006 | EF468567 | Domingues *et al*., 2007 |
| 3 | SP103 | 2006 | EF468568 | Domingues *et al*., 2007 |
| 4 | SP104 | 2006 | EF468569 | Domingues *et al*., 2007 |
|  | CPN266 | 2014 | MG992746 | present work |
| 5 | SP106 | 2006 | EF468571 | Domingues et al., 2007 |
| 6 | SP107 | 2006 | EF468572 | Domingues et al., 2007 |
| 7 | SP108 | 2006 | EF468573 | Domingues et al., 2007 |
|  | SP105 | 2006 | EF468570 | Domingues et al., 2007 |
|  | CPN103 | 2011 | MG992656 | present work |
| 8 | SP109 | 2006 | EF468574 | Domingues et al., 2007 |
| 9 | SP110 | 2006 | EF468575 | Domingues et al., 2007 |
|  | N272 | 2014 | MG992751 | present work |
| 10 | SP111 | 2006 | EF468576 | Domingues et al., 2007 |
| 11 | SP112 | 2006 | EF468577 | Domingues et al., 2007 |
| 12 | SP113 | 2006 | EF468578 | Domingues et al., 2007 |
| 13 | SP114 | 2006 | EF468579 | Domingues et al., 2007 |
| 14 | SP115 | 2006 | EF468580 | Domingues et al., 2007 |
| 15 | SP118 | 2006 | EF468581 | Domingues et al., 2007 |
|  | N239 | 2014 | MG992718 | present work |
| 16 | SP119 | 2006 | EF468582 | Domingues et al., 2007 |
| 17 | SP120 | 2006 | EF468583 | Domingues et al., 2007 |
| 18 | SP122 | 2006 | EF468584 | Domingues et al., 2007 |
| 19 | SP123 | 2006 | EF468585 | Domingues et al., 2007 |
| 20 | CPN1 | 2009 | MG992598 | present work |
| 21 | CPN2 | 2009 | MG992599 | present work |
|  | CPN48 | 2009 | MG992617 | present work |
| 22 | CPN3 | 2009 | MG992600 | present work |
| 23 | CPN4 | 2009 | MG992601 | present work |
|  | CPN5 | 2009 | MG992602 | present work |
|  | CPN34 | 2009 | MG992609 | present work |
|  | CPN51 | 2009 | MG992620 | present work |
|  | CPN53 | 2009 | MG992622 | present work |
|  | CPN60 | 2011 | MG992629 | present work |
|  | CPN96 | 2011 | MG992654 | present work |
|  | CPN109 | 2011 | MG992662 | present work |
|  | CPN194 | 2014 | MG992679 | present work |
|  | CPN204 | 2014 | MG992688 | present work |
|  | CPN222 | 2014 | MG992705 | present work |
|  | CPN235 | 2014 | MG992715 | present work |
|  | CPN252 | 2014 | MG992731 | present work |
| 24 | CPN6 | 2009 | MG992603 | present work |
| 25 | CPN7 | 2009 | MG992604 | present work |
| 26 | CPN8 | 2009 | MG992605 | present work |
|  | CPN10 | 2009 | MG992607 | present work |
| 27 | CPN9 | 2009 | MG992606 | present work |
| 28 | CPN11 | 2009 | MG992608 | present work |
| 29 | CPN35 | 2009 | MG992610 | present work |
| 30 | CPN38 | 2009 | MG992611 | present work |
| 31 | CPN43 | 2009 | MG992612 | present work |
| 32 | CPN44 | 2009 | MG992613 | present work |
|  | CPN184 | 2014 | MG992670 | present work |
|  | CPN238 | 2014 | MG992717 | present work |
| 33 | CPN45 | 2009 | MG992614 | present work |
|  | CPN72 | 2011 | MG992638 | present work |
|  | CPN245 | 2014 | MG992724 | present work |
| 34 | CPN46 | 2009 | MG992615 | present work |
|  | CPN227 | 2014 | MG992710 | present work |
| 35 | CPN47 | 2009 | MG992616 | present work |
| 36 | CPN49 | 2009 | MG992618 | present work |
|  | CPN270 | 2014 | MG992749 | present work |
| 37 | CPN50 | 2009 | MG992619 | present work |
|  | CPN62 | 2011 | MG992631 | present work |
| 38 | CPN52 | 2009 | MG992621 | present work |
|  | CPN178 | 2014 | MG992664 | present work |
| 39 | CPN54 | 2009 | MG992623 | present work |
|  | CPN226 | 2014 | MG992709 | present work |
| 40 | CPN55 | 2009 | MG992624 | present work |
| 41 | CPN56 | 2009 | MG992625 | present work |
| 42 | CPN57 | 2009 | MG992626 | present work |
| 43 | CPN58 | 2009 | MG992627 | present work |
| 44 | CPN59 | 2011 | MG992628 | present work |
|  | CPN105 | 2011 | MG992658 | present work |
| 45 | CPN61 | 2011 | MG992630 | present work |
| 46 | CPN64 | 2011 | MG992632 | present work |
| 47 | CPN65 | 2011 | MG992633 | present work |
| 48 | CPN66 | 2011 | MG992634 | present work |
| 49 | CPN69 | 2011 | MG992635 | present work |
| 50 | CPN70 | 2011 | MG992636 | present work |
| 51 | CPN71 | 2011 | MG992637 | present work |
|  | CPN85 | 2009 | MG992649 | present work |
| 52 | CPN73 | 2009 | MG992639 | present work |
| 53 | CPN74 | 2009 | MG992640 | present work |
|  | CPN79 | 2009 | MG992644 | present work |
| 54 | CPN75 | 2009 | MG992641 | present work |
| 55 | CPN76 | 2009 | MG992642 | present work |
| 56 | CPN77 | 2009 | MG992643 | present work |
|  | CPN91 | 2011 | MG992650 | present work |
| 57 | CPN81 | 2009 | MG992645 | present work |
|  | CPN83 | 2009 | MG992647 | present work |
|  | CPN224 | 2014 | MG992707 | present work |
| 58 | CPN82 | 2009 | MG992646 | present work |
| 59 | CPN84 | 2009 | MG992648 | present work |
| 60 | CPN93 | 2011 | MG992651 | present work |
|  | CPN94 | 2011 | MG992652 | present work |
| 61 | CPN95 | 2011 | MG992653 | present work |
|  | CPN247 | 2014 | MG992726 | present work |
|  | CPN274 | 2014 | MG992753 | present work |
|  | CPN279 | 2014 | MG992757 | present work |
| 62 | CPN97 | 2011 | MG992655 | present work |
| 63 | CPN104 | 2011 | MG992657 | present work |
| 64 | CPN106 | 2011 | MG992659 | present work |
| 65 | CPN107 | 2011 | MG992660 | present work |
|  | CPN177 | 2014 | MG992663 | present work |
|  | CPN213 | 2014 | MG992697 | present work |
|  | CPN221 | 2014 | MG992704 | present work |
| 66 | CPN108 | 2011 | MG992661 | present work |
|  | CPN193 | 2014 | MG992678 | present work |
|  | CPN196 | 2014 | MG992681 | present work |
|  | CPN223 | 2014 | MG992706 | present work |
| 67 | CPN179 | 2014 | MG992665 | present work |
|  | CPN271 | 2014 | MG992750 | present work |
| 68 | CPN180 | 2014 | MG992666 | present work |
| 69 | CPN181 | 2014 | MG992667 | present work |
| 70 | CPN182 | 2014 | MG992668 | present work |
|  | CPN251 | 2014 | MG992730 | present work |
| 71 | CPN183 | 2014 | MG992669 | present work |
|  | CPN260 | 2014 | MG992740 | present work |
| 72 | CPN185 | 2014 | MG992671 | present work |
| 73 | CPN186 | 2014 | MG992672 | present work |
| 74 | CPN187 | 2014 | MG992673 | present work |
|  | CPN242 | 2014 | MG992721 | present work |
| 75 | CPN188 | 2014 | MG992674 | present work |
| 76 | CPN189 | 2014 | MG992675 | present work |
| 77 | CPN191 | 2014 | MG992676 | present work |
| 78 | CPN192 | 2014 | MG992677 | present work |
| 79 | CPN195 | 2014 | MG992680 | present work |
|  | CPN231 | 2014 | MG992712 | present work |
|  | CPN264 | 2014 | MG992743 | present work |
| 80 | CPN197 | 2014 | MG992682 | present work |
| 81 | CPN198 | 2014 | MG992683 | present work |
| 82 | CPN199 | 2014 | MG992684 | present work |
|  | CPN277 | 2014 | MG992755 | present work |
| 83 | CPN201 | 2014 | MG992685 | present work |
| 84 | CPN202 | 2014 | MG992686 | present work |
| 85 | CPN203 | 2014 | MG992687 | present work |
| 86 | CPN205 | 2014 | MG992689 | present work |
| 87 | CPN206 | 2014 | MG992690 | present work |
|  | CPN208 | 2014 | MG992692 | present work |
| 88 | CPN207 | 2014 | MG992691 | present work |
| 89 | CPN209 | 2014 | MG992693 | present work |
| 90 | CPN210 | 2014 | MG992694 | present work |
| 91 | CPN211 | 2014 | MG992695 | present work |
|  | CPN218 | 2014 | MG992701 | present work |
| 92 | CPN212 | 2014 | MG992696 | present work |
| 93 | CPN215 | 2014 | MG992698 | present work |
| 94 | CPN216 | 2014 | MG992699 | present work |
| 95 | CPN217 | 2014 | MG992700 | present work |
| 96 | CPN219 | 2014 | MG992702 | present work |
| 97 | CPN220 | 2014 | MG992703 | present work |
| 98 | CPN225 | 2014 | MG992708 | present work |
| 99 | CPN228 | 2014 | MG992711 | present work |
| 100 | CPN236 | 2014 | MG992713 | present work |
| 101 | CPN234 | 2014 | MG992714 | present work |
| 102 | CPN237 | 2014 | MG992716 | present work |
| 103 | CPN241 | 2014 | MG992720 | present work |
| 104 | CPN243 | 2014 | MG992722 | present work |
| 105 | CPN244 | 2014 | MG992723 | present work |
|  | CPN246 | 2014 | MG992725 | present work |
|  | CPN273 | 2014 | MG992752 | present work |
| 106 | CPN248 | 2014 | MG992727 | present work |
| 107 | CPN249 | 2014 | MG992728 | present work |
| 108 | CPN250 | 2014 | MG992729 | present work |
| 109 | CPN253 | 2014 | MG992732 | present work |
| 110 | CPN254 | 2014 | MG992733 | present work |
| 111 | CPN255 | 2014 | MG992734 | present work |
| 112 | CPN256 | 2014 | MG992735 | present work |
| 113 | CPN257 | 2014 | MG992736 | present work |
| 114 | CPN258 | 2014 | MG992737 | present work |
| 115 | CPN259 | 2014 | MG992738 | present work |
| 116 | CPN261 | 2014 | MG992739 | present work |
| 117 | CPN262 | 2014 | MG992741 | present work |
| 118 | CPN263 | 2014 | MG992742 | present work |
| 119 | CPN265 | 2014 | MG992744 | present work |
| 120 | CPN267 | 2014 | MG992746 | present work |
| 121 | CPN268 | 2014 | MG992747 | present work |
| 122 | CPN269 | 2014 | MG992748 | present work |
| 123 | CPN276 | 2014 | MG992754 | present work |
| 124 | CPN278 | 2014 | MG992756 | present work |
| 1 | SP100 | 2003 | EF467725 | Domingues et al., 2007 |
| 2 | SP101 | 2003 | EF467726 | Domingues et al., 2007 |
| 3 | SP102 | 2003 | EF467727 | Domingues et al., 2007 |
| 4 | SP103 | 2003 | EF467728 | Domingues et al., 2007 |
| 5 | SP104 | 2003 | EF467729 | Domingues et al., 2007 |
| 6 | SP105 | 2003 | EF467730 | Domingues et al., 2007 |
| 7 | SP106 | 2003 | EF467731 | Domingues et al., 2007 |
| 8 | SP107 | 2003 | EF467732 | Domingues et al., 2007 |
| 9 | SP108 | 2003 | EF467733 | Domingues et al., 2007 |
| 10 | SP109 | 2003 | EF467734 | Domingues et al., 2007 |
|  | CPN273 | 2014 | MG992882 | present work |
| 11 | SP110 | 2003 | EF467735 | Domingues et al., 2007 |
| 12 | SP111 | 2003 | EF467736 | Domingues et al., 2007 |
|  | CPN187 | 2014 | MG992821 | present work |
| 13 | SP112 | 2003 | EF467737 | Domingues et al., 2007 |
| 14 | SP113 | 2003 | EF467738 | Domingues et al., 2007 |
| 15 | SP114 | 2003 | EF467739 | Domingues et al., 2007 |
| 16 | SP115 | 2003 | EF467740 | Domingues et al., 2007 |
| 17 | SP118 | 2003 | EF467741 | Domingues et al., 2007 |
| 18 | SP119 | 2003 | EF467742 | Domingues et al., 2007 |
| 19 | SP120 | 2003 | EF467743 | Domingues et al., 2007 |
| 20 | SP122 | 2003 | EF467744 | Domingues et al., 2007 |
| 21 | CPN1 | 2009 | MG992758 | Present work |
|  | CPN103 | 2011 | MG992807 | Present work |
|  | CPN201 | 2014 | MG992833 | Present work |
| 22 | CPN2 | 2009 | MG992759 | Present work |
| 23 | CPN3 | 2009 | MG992760 | Present work |
| 24 | CPN5 | 2009 | MG992761 | Present work |
| 25 | CPN7 | 2009 | MG992762 | Present work |
|  | CPN49 | 2009 | MG992774 | Present work |
| 26 | CPN97 | 2011 | MG992806 | Present work |
| 27 | CPN8 | 2009 | MG992763 | Present work |
| 28 | CPN11 | 2009 | MG992764 | Present work |
| 29 | CPN34 | 2009 | MG992765 | Present work |
| 30 | CPN35 | 2009 | MG992766 | Present work |
| 31 | CPN38 | 2009 | MG992767 | Present work |
| 32 | CPN43 | 2009 | MG992768 | Present work |
| 33 | CPN44 | 2009 | MG992769 | Present work |
| 34 | CPN45 | 2009 | MG992770 | Present work |
| 35 | CPN46 | 2009 | MG992771 | Present work |
| 36 | CPN47 | 2009 | MG992772 | Present work |
| 37 | CPN48 | 2009 | MG992773 | Present work |
| 38 | CPN50 | 2009 | MG992775 | Present work |
| 39 | CPN51 | 2009 | MG992776 | Present work |
| 40 | CPN52 | 2009 | MG992777 | Present work |
| 41 | CPN53 | 2009 | MG992778 | Present work |
| 42 | CPN54 | 2009 | MG992779 | Present work |
|  | CPN104 | 2011 | MG992808 | Present work |
| 43 | CPN55 | 2009 | MG992780 | Present work |
|  | CPN74 | 2009 | MG992792 | Present work |
|  | CPN258 | 2014 | MG992869 | Present work |
| 44 | CPN56 | 2009 | MG992781 | Present work |
| 45 | CPN57 | 2009 | MG992782 | Present work |
|  | CPN178 | 2014 | MG992813 | Present work |
| 46 | CPN58 | 2009 | MG992783 | Present work |
| 47 | CPN61 | 2011 | MG992784 | Present work |
| 48 | CPN62 | 2011 | MG992785 | Present work |
|  | CPN193 | 2014 | MG992827 | Present work |
|  | CPN239 | 2014 | MG992854 | Present work |
| 49 | CPN63 | 2011 | MG992786 | Present work |
| 50 | CPN64 | 2011 | MG992787 | Present work |
| 51 | CPN65 | 2011 | MG992788 | Present work |
| 52 | CPN66 | 2011 | MG992789 | Present work |
|  | CPN75 | 2009 | MG992793 | Present work |
|  | CPN179 | 2014 | MG992814 | Present work |
| 53 | CPN70 | 2011 | MG992790 | Present work |
| 54 | CPN72 | 2011 | MG992791 | Present work |
| 55 | CPN76 | 2009 | MG992794 | Present work |
| 56 | CPN77 | 2009 | MG992795 | Present work |
|  | CPN245 | 2014 | MG992858 | Present work |
| 57 | CPN78 | 2009 | MG992796 | Present work |
| 58 | CPN79 | 2009 | MG992797 | Present work |
| 59 | CPN81 | 2009 | MG992798 | Present work |
| 60 | CPN82 | 2014 | MG992799 | Present work |
| 61 | CPN83 | 2014 | MG992800 | Present work |
| 62 | CPN84 | 2014 | MG992801 | Present work |
| 63 | CPN85 | 2014 | MG992802 | Present work |
|  | CPN249 | 2014 | MG992861 | Present work |
|  | CPN257 | 2014 | MG992868 | Present work |
| 64 | CPN91 | 2014 | MG992803 | Present work |
| 65 | CPN94 | 2014 | MG992804 | Present work |
| 66 | CPN96 | 2014 | MG992805 | Present work |
| 67 | CPN105 | 2014 | MG992809 | Present work |
| 68 | CPN106 | 2014 | MG992810 | Present work |
| 69 | CPN107 | 2014 | MG992811 | Present work |
| 70 | CPN108 | 2014 | MG992812 | Present work |
| 71 | CPN180 | 2014 | MG992815 | Present work |
| 72 | CPN181 | 2014 | MG992816 | Present work |
| 73 | CPN182 | 2014 | MG992817 | Present work |
|  | CPN207 | 2014 | MG992839 | Present work |
|  | CPN224 | 2014 | MG992849 | Present work |
|  | CPN238 | 2014 | MG992853 | Present work |
| 74 | CPN183 | 2014 | MG992818 | Present work |
| 75 | CPN184 | 2014 | MG992819 | Present work |
|  | CPN195 | 2014 | MG992829 | Present work |
| 76 | CPN185 | 2014 | MG992820 | Present work |
| 77 | CPN188 | 2014 | MG992822 | Present work |
|  | CPN222 | 2014 | MG992847 | Present work |
| 78 | CPN189 | 2014 | MG992823 | Present work |
| 79 | CPN190 | 2014 | MG992824 | Present work |
|  | CPN223 | 2014 | MG992848 | Present work |
| 80 | CPN191 | 2014 | MG992825 | Present work |
| 81 | CPN192 | 2014 | MG992826 | Present work |
| 82 | CPN194 | 2014 | MG992828 | Present work |
| 83 | CPN196 | 2014 | MG992830 | Present work |
| 84 | CPN199 | 2014 | MG992831 | Present work |
| 85 | CPN200 | 2014 | MG992832 | Present work |
| 86 | CPN202 | 2014 | MG992834 | Present work |
| 87 | CPN203 | 2014 | MG992835 | Present work |
| 88 | CPN204 | 2014 | MG992836 | Present work |
| 89 | CPN205 | 2014 | MG992837 | Present work |
| 90 | CPN206 | 2014 | MG992838 | Present work |
| 91 | CPN210 | 2014 | MG992840 | Present work |
| 92 | CPN211 | 2014 | MG992841 | Present work |
| 93 | CPN212 | 2014 | MG992842 | Present work |
| 94 | CPN213 | 2014 | MG992843 | Present work |
| 95 | CPN218 | 2014 | MG992844 | Present work |
| 96 | CPN219 | 2014 | MG992845 | Present work |
| 97 | CPN221 | 2014 | MG992846 | Present work |
| 98 | CPN225 | 2014 | MG992850 | Present work |
| 99 | CPN228 | 2014 | MG992851 | Present work |
| 100 | CPN237 | 2014 | MG992852 | Present work |
| 101 | CPN240 | 2014 | MG992855 | Present work |
| 102 | CPN242 | 2014 | MG992856 | Present work |
| 103 | CPN244 | 2014 | MG992857 | Present work |
| 104 | CPN246 | 2014 | MG992859 | Present work |
| 105 | CPN248 | 2014 | MG992860 | Present work |
| 106 | CPN250 | 2014 | MG992862 | Present work |
| 107 | CPN252 | 2014 | MG992863 | Present work |
| 108 | CPN253 | 2014 | MG992864 | Present work |
| 109 | CPN254 | 2014 | MG992865 | Present work |
| 110 | CPN255 | 2014 | MG992866 | Present work |
| 111 | CPN256 | 2014 | MG992867 | Present work |
| 112 | CPN259 | 2014 | MG992870 | Present work |
| 113 | CPN262 | 2014 | MG992871 | Present work |
| 114 | CPN263 | 2014 | MG992872 | Present work |
| 115 | CPN264 | 2014 | MG992873 | Present work |
| 116 | CPN265 | 2014 | MG992874 | Present work |
| 117 | CPN266 | 2014 | MG992875 | Present work |
| 118 | CPN267 | 2014 | MG992876 | Present work |
| 119 | CPN268 | 2014 | MG992877 | Present work |
|  | CPN269 | 2014 | MG992878 | Present work |
| 120 | CPN270 | 2014 | MG992879 | Present work |
| 121 | CPN271 | 2014 | MG992880 | Present work |
| 122 | CPN272 | 2014 | MG992881 | Present work |
| 123 | CPN274 | 2014 | MG992883 | Present work |
| 124 | CPN275 | 2014 | MG992884 | Present work |
| 125 | CPN276 | 2014 | MG992885 | Present work |
| 126 | CPN277 | 2014 | MG992886 | Present work |
| 127 | CPN278 | 2014 | MG992887 | Present work |
| 128 | CPN279 | 2014 | MG992888 | Present work |
|  |  |  |  |  |

Table S2 – Sampling periods, specimens and Genebank Accession Numbers for CR and S7 of *Atherina presbyter*.

|  |  |  |  |  |
| --- | --- | --- | --- | --- |
| Haplotype number | Specimen | Sampling year | Accession number | Work |
| 1 | CPN123 | 2014 | KR028696 | Francisco & Robalo, 2015 |
|  | CPN243 | 2014 | KR028774 | Francisco & Robalo, 2015 |
|  | CPS178 | 2012 | KR028694 | Francisco & Robalo, 2015 |
|  | CPS36 | 2013 | KR028553 | Francisco & Robalo, 2015 |
| 2 | FT12 | 2005 | DQ336742 | Francisco *et al.*, 2006a |
| 3 | CPN177 | 2014 | KR028707 | Francisco & Robalo, 2015 |
|  | CPN204 | 2014 | KR028736 | Francisco & Robalo, 2015 |
|  | CPN224 | 2014 | KR028755 | Francisco & Robalo, 2015 |
|  | CPN226 | 2014 | KR028757 | Francisco & Robalo, 2015 |
|  | CPN229 | 2014 | KR028760 | Francisco & Robalo, 2015 |
| 4 | SP1 | 2005 | EF611544 | Francisco *et al*., 2008 |
| 5 | CPN180 | 2014 | KR028710 | Francisco & Robalo, 2015 |
|  | FT18 | 2005 | DQ336744 | Francisco *et al.*, 2006a |
|  | CPN232 | 2014 | KR028763 | Francisco & Robalo, 2015 |
|  | CPN258 | 2014 | KR028788 | Francisco & Robalo, 2015 |
| 6 | CPS79 | 2013 | KR028595 | Francisco & Robalo, 2015 |
|  | CPS80 | 2013 | KR028596 | Francisco & Robalo, 2015 |
| 7 | CPN214 | 2014 | KR028745 | Francisco & Robalo, 2015 |
| 8 | CPN190 | 2014 | KR028722 | Francisco & Robalo, 2015 |
| 9 | CPN173 | 2014 | KR028703 | Francisco & Robalo, 2015 |
| 10 | CPN189 | 2014 | KR028721 | Francisco & Robalo, 2015 |
|  | FT10 | 2005 | DQ336736 | Francisco *et al.*, 2006a |
|  | CPN196 | 2014 | KR028728 | Francisco & Robalo, 2015 |
|  | CPN218 | 2014 | KR028749 | Francisco & Robalo, 2015 |
|  | CPN230 | 2014 | KR028761 | Francisco & Robalo, 2015 |
|  | CPN250 | 2014 | KR028781 | Francisco & Robalo, 2015 |
|  | CPS114 | 2012 | KR028629 | Francisco & Robalo, 2015 |
|  | CPS145 | 2012 | KR028660 | Francisco & Robalo, 2015 |
|  | CPS151 | 2012 | KR028666 | Francisco & Robalo, 2015 |
|  | CPS43 | 2013 | KR028560 | Francisco & Robalo, 2015 |
|  | CPS58 | 2013 | KR028575 | Francisco & Robalo, 2015 |
| 11 | CPN207 | 2014 | KR028739 | Francisco & Robalo, 2015 |
| 12 | FT13 | 2005 | DQ336739 | Francisco *et al.*, 2006a |
| 13 | CPN249 | 2014 | KR028780 | Francisco & Robalo, 2015 |
|  | FT15 | 2005 | DQ336740 | Francisco *et al.*, 2006a |
| 14 | CPS106 | 2012 | KR028622 | Francisco & Robalo, 2015 |
| 15 | CPS100 | 2012 | KR028616 | Francisco & Robalo, 2015 |
| 16 | CPS96 | 2012 | KR028613 | Francisco & Robalo, 2015 |
| 17 | CPS51 | 2013 | KR028568 | Francisco & Robalo, 2015 |
| 18 | FT22 | 2005 | DQ336724 | Francisco *et al.*, 2006a |
| 19 | FT20 | 2005 | DQ336722 | Francisco *et al.*, 2006a |
|  | FT4 | 2005 | DQ336721 | Francisco *et al.*, 2006a |
|  | CPN170 | 2014 | KR028700 | Francisco & Robalo, 2015 |
|  | CPN228 | 2014 | KR028759 | Francisco & Robalo, 2015 |
|  | CPS141 | 2012 | KR028656 | Francisco & Robalo, 2015 |
|  | CPS148 | 2012 | KR028663 | Francisco & Robalo, 2015 |
|  | CPS32 | 2013 | KR028549 | Francisco & Robalo, 2015 |
|  | CPS64 | 2013 | KR028581 | Francisco & Robalo, 2015 |
| 20 | CPS126 | 2012 | KR028641 | Francisco & Robalo, 2015 |
| 21 | CPS150 | 2012 | KR028665 | Francisco & Robalo, 2015 |
| 22 | CPN175 | 2014 | KR028705 | Francisco & Robalo, 2015 |
|  | CPS128 | 2012 | KR028643 | Francisco & Robalo, 2015 |
|  | CPS136 | 2012 | KR028651 | Francisco & Robalo, 2015 |
| 23 | FT16 | 2005 | DQ336728 | Francisco *et al.*, 2006a |
| 24 | CPN192 | 2014 | KR028724 | Francisco & Robalo, 2015 |
| 25 | CPS88 | 2013 | KR028604 | Francisco & Robalo, 2015 |
| 26 | CPS108 | 2012 | KR028624 | Francisco & Robalo, 2015 |
|  | CPS175 | 2012 | KR028690 | Francisco & Robalo, 2015 |
| 27 | CPS50 | 2013 | KR028567 | Francisco & Robalo, 2015 |
| 28 | CPN203 | 2014 | KR028735 | Francisco & Robalo, 2015 |
| 29 | CPN216 | 2014 | KR028747 | Francisco & Robalo, 2015 |
| 30 | CPS161 | 2012 | KR028676 | Francisco & Robalo, 2015 |
| 31 | CPN206 | 2014 | KR028738 | Francisco & Robalo, 2015 |
| 32 | CPN167 | 2014 | KR028697 | Francisco & Robalo, 2015 |
| 33 | CPS124 | 2012 | KR028639 | Francisco & Robalo, 2015 |
| 34 | CPS134 | 2012 | KR028649 | Francisco & Robalo, 2015 |
|  | CPS140 | 2012 | KR028655 | Francisco & Robalo, 2015 |
|  | CPS155 | 2012 | KR028670 | Francisco & Robalo, 2015 |
|  | CPS98 | 2012 | KR028615 | Francisco & Robalo, 2015 |
| 35 | CPN172 | 2014 | KR028702 | Francisco & Robalo, 2015 |
| 36 | CPS137 | 2012 | KR028652 | Francisco & Robalo, 2015 |
| 37 | CPS154 | 2012 | KR028669 | Francisco & Robalo, 2015 |
| 38 | CPS119 | 2012 | KR028634 | Francisco & Robalo, 2015 |
| 39 | CPN239 | 2014 | KR028770 | Francisco & Robalo, 2015 |
|  | CPS109 | 2012 | KR028625 | Francisco & Robalo, 2015 |
|  | CPS176 | 2012 | KR028691 | Francisco & Robalo, 2015 |
| 40 | FT17 | 2005 | DQ336715 | Francisco *et al.*, 2006a |
| 41 | CPN182 | 2014 | KR028713 | Francisco & Robalo, 2015 |
|  | CPN219 | 2014 | KR028750 | Francisco & Robalo, 2015 |
|  | CPS103 | 2012 | KR028619 | Francisco & Robalo, 2015 |
| 42 | CPS130 | 2012 | KR028645 | Francisco & Robalo, 2015 |
| 43 | CPS31 | 2013 | KR028548 | Francisco & Robalo, 2015 |
| 44 | CPS117 | 2012 | KR028632 | Francisco & Robalo, 2015 |
| 45 | FT9 | 2005 | DQ336717 | Francisco *et al.*, 2006a |
| 46 | CPN187 | 2014 | KR028719 | Francisco & Robalo, 2015 |
| 47 | CPN241 | 2014 | KR028772 | Francisco & Robalo, 2015 |
| 48 | CPS129 | 2012 | KR028644 | Francisco & Robalo, 2015 |
|  | CPS49 | 2013 | KR028566 | Francisco & Robalo, 2015 |
| 49 | CPN183 | 2014 | KR028715 | Francisco & Robalo, 2015 |
| 50 | FT14 | 2005 | DQ336716 | Francisco *et al.*, 2006a |
| 51 | CPN197 | 2014 | KR028729 | Francisco & Robalo, 2015 |
|  | CPS171 | 2012 | KR028686 | Francisco & Robalo, 2015 |
|  | CPS172 | 2012 | KR028687 | Francisco & Robalo, 2015 |
| 52 | CPS132 | 2012 | KR028647 | Francisco & Robalo, 2015 |
| 53 | CPS62 | 2013 | KR028579 | Francisco & Robalo, 2015 |
| 54 | CPN169 | 2014 | KR028699 | Francisco & Robalo, 2015 |
|  | CPS163 | 2012 | KR028678 | Francisco & Robalo, 2015 |
|  | CPS45 | 2013 | KR028562 | Francisco & Robalo, 2015 |
|  | CPS59 | 2013 | KR028576 | Francisco & Robalo, 2015 |
|  | CPS99 | 2012 | KR028616 | Francisco & Robalo, 2015 |
| 55 | CPS156 | 2012 | KR028671 | Francisco & Robalo, 2015 |
| 56 | Lab5 | 2005 | EF611549 | Francisco *et al*., 2008 |
| 57 | FT7 | 2005 | DQ336718 | Francisco *et al.*, 2006a |
| 58 | FT19 | 2005 | DQ336719 | Francisco *et al.*, 2006a |
| 59 | CPS40 | 2013 | KR028557 | Francisco & Robalo, 2015 |
| 60 | CPN184 | 2014 | KR028716 | Francisco & Robalo, 2015 |
| 61 | CPS69 | 2013 | KR028586 | Francisco & Robalo, 2015 |
| 62 | CPS30 | 2013 | KR028547 | Francisco & Robalo, 2015 |
| 63 | CPS121 | 2012 | KR028636 | Francisco & Robalo, 2015 |
| 64 | CPN222 | 2014 | KR028753 | Francisco & Robalo, 2015 |
| 65 | CPS57 | 2013 | KR028574 | Francisco & Robalo, 2015 |
| 66 | CPN176 | 2014 | KR028706 | Francisco & Robalo, 2015 |
|  | CPN252 | 2014 | KR028783 | Francisco & Robalo, 2015 |
|  | CPN253 | 2014 | KR028784 | Francisco & Robalo, 2015 |
|  | CPS84 | 2013 | KR028600 | Francisco & Robalo, 2015 |
| 67 | FT24 | 2005 | DQ336746 | Francisco *et al.*, 2006a |
| 68 | CPN185 | 2014 | KR028717 | Francisco & Robalo, 2015 |
| 69 | CPS56 | 2013 | KR028573 | Francisco & Robalo, 2015 |
| 70 | Lab1 | 2005 | EF611547 | Francisco *et al*., 2008 |
|  | Lab3 | 2005 | EU181428 | Francisco *et al*., 2008 |
| 71 | Lab2 | 2005 | EF611567 | Francisco *et al*., 2008 |
|  | CPS112 | 2012 | KR028627 | Francisco & Robalo, 2015 |
|  | CPS166 | 2012 | KR028681 | Francisco & Robalo, 2015 |
|  | CPS53 | 2013 | KR028570 | Francisco & Robalo, 2015 |
| 72 | FT11 | 2005 | DQ336747 | Francisco *et al.*, 2006a |
| 73 | CPS71 | 2013 | KR028588 | Francisco & Robalo, 2015 |
| 74 | FT5 | 2005 | DQ336758 | Francisco *et al.*, 2006a |
|  | FT23 | 2005 | DQ336760 | Francisco *et al.*, 2006a |
|  | FT2 | 2005 | DQ336759 | Francisco *et al.*, 2006a |
|  | Arr2 | 2005 | DQ336761 | Francisco *et al.*, 2006a |
|  | CPS97 | 2012 | KR028614 | Francisco & Robalo, 2015 |
|  | CPS95 | 2012 | KR028612 | Francisco & Robalo, 2015 |
|  | CPS89 | 2012 | KR028605 | Francisco & Robalo, 2015 |
|  | CPS82 | 2013 | KR028598 | Francisco & Robalo, 2015 |
|  | CPS42 | 2013 | KR028559 | Francisco & Robalo, 2015 |
|  | CPS39 | 2013 | KR028556 | Francisco & Robalo, 2015 |
|  | CPS179 | 2012 | KR028695 | Francisco & Robalo, 2015 |
|  | CPS144 | 2012 | KR028659 | Francisco & Robalo, 2015 |
|  | CPS125 | 2012 | KR028640 | Francisco & Robalo, 2015 |
|  | CPS116 | 2012 | KR028631 | Francisco & Robalo, 2015 |
|  | CPS113 | 2012 | KR028628 | Francisco & Robalo, 2015 |
|  | CPS107 | 2012 | KR028623 | Francisco & Robalo, 2015 |
|  | CPN221 | 2014 | KR028752 | Francisco & Robalo, 2015 |
|  | CPN240 | 2014 | KR028771 | Francisco & Robalo, 2015 |
|  | CPN236 | 2014 | KR028767 | Francisco & Robalo, 2015 |
|  | CPN213 | 2014 | KR028744 | Francisco & Robalo, 2015 |
|  | CPN178 | 2014 | KR028708 | Francisco & Robalo, 2015 |
|  | CPN122 | 2014 | KR028695 | Francisco & Robalo, 2015 |
| 75 | Lab7 | 2005 | EF611551 | Francisco *et al*., 2008 |
| 76 | CPS33 | 2013 | KR028550 | Francisco & Robalo, 2015 |
| 77 | CPS48 | 2013 | KR028565 | Francisco & Robalo, 2015 |
| 78 | CPS160 | 2012 | KR028675 | Francisco & Robalo, 2015 |
| 79 | CPS149 | 2012 | KR028664 | Francisco & Robalo, 2015 |
| 80 | CPN186 | 2014 | KR028718 | Francisco & Robalo, 2015 |
| 81 | CPN188 | 2014 | KR028720 | Francisco & Robalo, 2015 |
|  | CPN238 | 2014 | KR028768 | Francisco & Robalo, 2015 |
|  | CPN251 | 2014 | KR028782 | Francisco & Robalo, 2015 |
|  | CPS168 | 2012 | KR028683 | Francisco & Robalo, 2015 |
|  | CPS37 | 2013 | KR028554 | Francisco & Robalo, 2015 |
| 82 | CPN255 | 2014 | KT159714 | Francisco & Robalo, 2015 |
| 83 | CPS162 | 2012 | KR028677 | Francisco & Robalo, 2015 |
| 84 | CPN237 | 2014 | KR028768 | Francisco & Robalo, 2015 |
| 85 | CPS90 | 2012 | KR028606 | Francisco & Robalo, 2015 |
| 86 | CPN254 | 2014 | KR028780 | Francisco & Robalo, 2015 |
| 87 | CPN181 | 2014 | KR028711 | Francisco & Robalo, 2015 |
|  | CPS159 | 2012 | KR028674 | Francisco & Robalo, 2015 |
| 88 | CPS120 | 2012 | KR028635 | Francisco & Robalo, 2015 |
| 89 | CPS173 | 2012 | KR028688 | Francisco & Robalo, 2015 |
| 90 | CPN212 | 2014 | KR028743 | Francisco & Robalo, 2015 |
| 91 | CPS46 | 2013 | KR028563 | Francisco & Robalo, 2015 |
| 92 | CPN220 | 2014 | KR028751 | Francisco & Robalo, 2015 |
| 93 | CPS135 | 2012 | KR028650 | Francisco & Robalo, 2015 |
| 94 | CPS167 | 2012 | KR028682 | Francisco & Robalo, 2015 |
| 95 | CPN247 | 2014 | KR028778 | Francisco & Robalo, 2015 |
|  | CPS87 | 2013 | KR028607 | Francisco & Robalo, 2015 |
| 96 | CPS38 | 2013 | KR028555 | Francisco & Robalo, 2015 |
| 97 | CPN244 | 2014 | KR028775 | Francisco & Robalo, 2015 |
| 98 | CPS27 | 2013 | KR028544 | Francisco & Robalo, 2015 |
| 99 | CPS70 | 2013 | KR028587 | Francisco & Robalo, 2015 |
| 100 | CPS76 | 2013 | KR028593 | Francisco & Robalo, 2015 |
| 101 | CPN233 | 2014 | KR028760 | Francisco & Robalo, 2015 |
| 102 | CPS92 | 2012 | KR028608 | Francisco & Robalo, 2015 |
| 103 | CPN195 | 2014 | KR028727 | Francisco & Robalo, 2015 |
| 104 | CPS139 | 2012 | KR028654 | Francisco & Robalo, 2015 |
| 105 | CPS72 | 2013 | KR028589 | Francisco & Robalo, 2015 |
| 106 | CPN225 | 2014 | KR028756 | Francisco & Robalo, 2015 |
|  | CPS152 | 2012 | KR028667 | Francisco & Robalo, 2015 |
| 107 | CPS127 | 2012 | KR028642 | Francisco & Robalo, 2015 |
| 108 | CPN234 | 2014 | KR028765 | Francisco & Robalo, 2015 |
| 109 | CPN235 | 2014 | KR028766 | Francisco & Robalo, 2015 |
| 110 | CPS111 | 2012 | KR028626 | Francisco & Robalo, 2015 |
|  | CPS158 | 2012 | KR028673 | Francisco & Robalo, 2015 |
| 111 | CPN191 | 2014 | KR028723 | Francisco & Robalo, 2015 |
| 112 | CPN256 | 2014 | KR028786 | Francisco & Robalo, 2015 |
| 113 | CPS29 | 2013 | KR028546 | Francisco & Robalo, 2015 |
| 114 | CPS35 | 2013 | KR028552 | Francisco & Robalo, 2015 |
| 115 | CPS94 | 2012 | KR028610 | Francisco & Robalo, 2015 |
| 116 | SP3 | 2005 | EF611546 | Francisco *et al*., 2008 |
| 117 | CPN174 | 2014 | KR028704 | Francisco & Robalo, 2015 |
| 118 | CPN182_1 | 2014 | KR028714 | Francisco & Robalo, 2015 |
| 119 | CPS81 | 2013 | KR028597 | Francisco & Robalo, 2015 |
| 120 | CPS34 | 2013 | KR028551 | Francisco & Robalo, 2015 |
|  | CPS66 | 2013 | KR028583 | Francisco & Robalo, 2015 |
| 121 | CPS83 | 2013 | KR028599 | Francisco & Robalo, 2015 |
| 122 | CPN168 | 2014 | KR028698 | Francisco & Robalo, 2015 |
|  | CPN199 | 2014 | KR028731 | Francisco & Robalo, 2015 |
|  | CPN201 | 2014 | KR028733 | Francisco & Robalo, 2015 |
|  | CPN210 | 2014 | KR028742 | Francisco & Robalo, 2015 |
|  | CPN215 | 2014 | KR028746 | Francisco & Robalo, 2015 |
|  | CPN231 | 2014 | KR028762 | Francisco & Robalo, 2015 |
|  | CPN242 | 2014 | KR028773 | Francisco & Robalo, 2015 |
|  | CPN245 | 2014 | KR028776 | Francisco & Robalo, 2015 |
|  | CPN246 | 2014 | KR028777 | Francisco & Robalo, 2015 |
|  | CPN248 | 2014 | KR028779 | Francisco & Robalo, 2015 |
|  | CPS123 | 2012 | KR028638 | Francisco & Robalo, 2015 |
|  | CPS131 | 2012 | KR028646 | Francisco & Robalo, 2015 |
|  | CPS146 | 2012 | KR028661 | Francisco & Robalo, 2015 |
|  | CPS174 | 2012 | KR028689 | Francisco & Robalo, 2015 |
|  | CPS28 | 2013 | KR028545 | Francisco & Robalo, 2015 |
|  | CPS60 | 2013 | KR028577 | Francisco & Robalo, 2015 |
|  | CPS61 | 2013 | KR028578 | Francisco & Robalo, 2015 |
| 123 | CPS52 | 2013 | KR028569 | Francisco & Robalo, 2015 |
| 124 | CPS101 | 2012 | KR028617 | Francisco & Robalo, 2015 |
| 125 | CPN202 | 2014 | KR028734 | Francisco & Robalo, 2015 |
|  | CPS93 | 2012 | KR028609 | Francisco & Robalo, 2015 |
| 126 | CPS170 | 2012 | KR028685 | Francisco & Robalo, 2015 |
| 127 | CPS55 | 2013 | KR028572 | Francisco & Robalo, 2015 |
| 128 | CPN257 | 2014 | KR028787 | Francisco & Robalo, 2015 |
|  | CPS153 | 2012 | KR028668 | Francisco & Robalo, 2015 |
|  | CPS63 | 2013 | KR028580 | Francisco & Robalo, 2015 |
|  | CPS77 | 2013 | KR028594 | Francisco & Robalo, 2015 |
| 129 | CPS157 | 2012 | KR028672 | Francisco & Robalo, 2015 |
|  | Lab4 | 2005 | EF611548 | Francisco *et al*., 2008 |
| 130 | CPS86 | 2013 | KR028602 | Francisco & Robalo, 2015 |
| 131 | CPS164 | 2012 | KR028679 | Francisco & Robalo, 2015 |
| 132 | Lab6 | 2005 | EF611550 | Francisco *et al*., 2008 |
| 133 | CPS122 | 2012 | KR028637 | Francisco & Robalo, 2015 |
|  | CPS169 | 2012 | KR028684 | Francisco & Robalo, 2015 |
| 134 | CPS143 | 2012 | KR028658 | Francisco & Robalo, 2015 |
| 135 | CPS26 | 2013 | KR028543 | Francisco & Robalo, 2015 |
| 136 | CPS104 | 2012 | KR028620 | Francisco & Robalo, 2015 |
|  | CPS118 | 2012 | KR028633 | Francisco & Robalo, 2015 |
| 137 | CPS65 | 2013 | KR028582 | Francisco & Robalo, 2015 |
| 138 | CPS75 | 2013 | KR028592 | Francisco & Robalo, 2015 |
| 139 | FT1 | 2005 | DQ336757 | Francisco *et al.*, 2006a |
| 140 | FT21 | 2005 | DQ336751 | Francisco *et al.*, 2006a |
| 141 | FT6 | 2005 | DQ336748 | Francisco *et al.*, 2006a |
| 142 | CPN171 | 2014 | KR028701 | Francisco & Robalo, 2015 |
|  | CPN179 | 2014 | KR028709 | Francisco & Robalo, 2015 |
|  | CPN194 | 2014 | KR028726 | Francisco & Robalo, 2015 |
|  | CPN217 | 2014 | KR028748 | Francisco & Robalo, 2015 |
|  | CPN223 | 2014 | KR028754 | Francisco & Robalo, 2015 |
|  | CPN227 | 2014 | KR028758 | Francisco & Robalo, 2015 |
|  | CPS115 | 2012 | KR028630 | Francisco & Robalo, 2015 |
|  | CPS138 | 2012 | KR028653 | Francisco & Robalo, 2015 |
|  | CPS165 | 2012 | KR028680 | Francisco & Robalo, 2015 |
|  | CPS54 | 2013 | KR028571 | Francisco & Robalo, 2015 |
|  | CPS85 | 2013 | KR028601 | Francisco & Robalo, 2015 |
| 143 | CPS133 | 2012 | KR028648 | Francisco & Robalo, 2015 |
| 144 | CPN205 | 2014 | KR028737 | Francisco & Robalo, 2015 |
| 145 | CPN193 | 2014 | KR028725 | Francisco & Robalo, 2015 |
| 146 | CPS177 | 2012 | KR028693 | Francisco & Robalo, 2015 |
| 147 | CPN181_1 | 2014 | KR028712 | Francisco & Robalo, 2015 |
|  | FT8 | 2005 | DQ336754 | Francisco *et al.*, 2006a |
|  | CPN200 | 2014 | KR028732 | Francisco & Robalo, 2015 |
|  | CPN208 | 2014 | KR028740 | Francisco & Robalo, 2015 |
|  | CPN209 | 2014 | KR028741 | Francisco & Robalo, 2015 |
|  | CPS142 | 2012 | KR028657 | Francisco & Robalo, 2015 |
|  | CPS44 | 2013 | KR028561 | Francisco & Robalo, 2015 |
|  | CPS68 | 2013 | KR028585 | Francisco & Robalo, 2015 |
| 148 | CPS102 | 2012 | KR028618 | Francisco & Robalo, 2015 |
| 149 | CPS147 | 2012 | KR028662 | Francisco & Robalo, 2015 |
| 150 | CPS67 | 2013 | KR028584 | Francisco & Robalo, 2015 |
|  | FT3 | 2005 | DQ336756 | Francisco *et al.*, 2006a |
| 151 | CPS105 | 2012 | KR028621 | Francisco & Robalo, 2015 |
| 152 | CPS41 | 2013 | KR028558 | Francisco & Robalo, 2015 |
| 153 | CPS74 | 2012 | KR028591 | Francisco & Robalo, 2015 |
| 154 | CPS47 | 2013 | KR028564 | Francisco & Robalo, 2015 |
|  | CPS73 | 2013 | KR028590 | Francisco & Robalo, 2015 |
|  | CPS91 | 2012 | KR028607 | Francisco & Robalo, 2015 |
| 155 | CPN198 | 2014 | KR028730 | Francisco & Robalo, 2015 |
| 1 | FT1 | 2005 | MH030878 | Present work |
|  | FT2 | 2005 | MH030879 | Present work |
|  | FT3 | 2005 | MH030880 | Present work |
|  | FT4 | 2005 | MH030881 | Present work |
|  | FT5 | 2005 | MH030882 | Present work |
|  | FT6 | 2005 | MH030883 | Present work |
|  | FT7 | 2005 | MH030884 | Present work |
|  | FT8 | 2005 | MH030885 | Present work |
|  | FT9 | 2005 | MH030886 | Present work |
|  | FT10 | 2005 | MH030887 | Present work |
|  | FT11 | 2005 | MH030888 | Present work |
|  | FT12a | 2005 | MH030889 | Present work |
|  | FT13a | 2005 | MH030891 | Present work |
|  | FT14 | 2005 | MH030893 | Present work |
|  | FT15 | 2005 | MH030894 | Present work |
|  | FT16 | 2005 | MH030895 | Present work |
|  | FT17 | 2005 | MH030896 | Present work |
|  | FT18 | 2005 | MH030897 | Present work |
|  | FT19 | 2005 | MH030898 | Present work |
|  | FT21 | 2005 | MH030899 | Present work |
|  | Lis1 | 2005 | MH030900 | Present work |
|  | Lis2 | 2005 | MH030901 | Present work |
|  | Lis3 | 2005 | MH030902 | Present work |
|  | Lis4 | 2005 | MH030903 | Present work |
|  | Lis5a | 2005 | MH030904 | Present work |
|  | Lis6 | 2005 | MH030906 | Present work |
|  | Lis7 | 2005 | MH030907 | Present work |
|  | Lis8 | 2005 | MH030908 | Present work |
|  | Lis9 | 2005 | MH030909 | Present work |
|  | Lis10a | 2005 | MH030910 | Present work |
|  | Lis11 | 2005 | MH030912 | Present work |
|  | Lis12 | 2005 | MH030913 | Present work |
|  | SP1 | 2005 | MH030914 | Present work |
|  | SP2 | 2005 | MH030915 | Present work |
|  | SP3 | 2005 | MH030916 | Present work |
|  | CPS25 | 2013 | MH030917 | Present work |
|  | CPS26a | 2013 | MH030918 | Present work |
|  | CPS28 | 2013 | MH030921 | Present work |
|  | CPS30 | 2013 | MH030924 | Present work |
|  | CPS31 | 2013 | MH030925 | Present work |
|  | CPS35 | 2013 | MH030929 | Present work |
|  | CPS37a | 2013 | MH030931 | Present work |
|  | CPS38 | 2013 | MH030933 | Present work |
|  | CPS40 | 2013 | MH030935 | Present work |
|  | CPS42 | 2013 | MH030938 | Present work |
|  | CPS43 | 2013 | MH030939 | Present work |
|  | CPS44 | 2013 | MH030940 | Present work |
|  | CPS45 | 2013 | MH030941 | Present work |
|  | CPS49a | 2013 | MH030945 | Present work |
|  | CPS50a | 2013 | MH030947 | Present work |
|  | CPS56 | 2013 | MH030955 | Present work |
|  | CPS57a | 2013 | MH030956 | Present work |
|  | CPS59b | 2013 | MH030961 | Present work |
|  | CPS62a | 2013 | MH030965 | Present work |
|  | CPS63b | 2013 | MH030968 | Present work |
|  | CPS67 | 2013 | MH030971 | Present work |
|  | CPS68a | 2013 | MH030972 | Present work |
|  | CPS69 | 2013 | MH030974 | Present work |
|  | CPS70 | 2013 | MH030975 | Present work |
|  | CPS71 | 2013 | MH030976 | Present work |
|  | CPS73 | 2013 | MH030979 | Present work |
|  | CPS78b | 2013 | MH030986 | Present work |
|  | CPS80a | 2013 | MH030988 | Present work |
|  | CPS82a | 2013 | MH030992 | Present work |
|  | CPS83a | 2013 | MH030994 | Present work |
|  | CPS84 | 2013 | MH030996 | Present work |
|  | CPS85 | 2013 | MH030997 | Present work |
|  | CPS86a | 2013 | MH030998 | Present work |
|  | CPS87 | 2013 | MH031000 | Present work |
|  | CPS88a | 2013 | MH031001 | Present work |
|  | CPS92a | 2012 | MH031006 | Present work |
|  | CPS95 | 2012 | MH031010 | Present work |
|  | CPS97 | 2012 | MH031012 | Present work |
|  | CPS98a | 2012 | MH031013 | Present work |
|  | CPS99 | 2012 | MH031015 | Present work |
|  | CPS100a | 2012 | MH031016 | Present work |
|  | CPS103a | 2012 | MH031022 | Present work |
|  | CPS104a | 2012 | MH031024 | Present work |
|  | CPS105a | 2012 | MH031026 | Present work |
|  | CPS106a | 2012 | MH031027 | Present work |
|  | CPS111a | 2012 | MH031035 | Present work |
|  | CPS112 | 2012 | MH031037 | Present work |
|  | CPS113 | 2012 | MH031038 | Present work |
|  | CPS114 | 2012 | MH031039 | Present work |
|  | CPS115 | 2012 | MH031040 | Present work |
|  | CPS116a | 2012 | MH031041 | Present work |
|  | CPS117a | 2012 | MH031043 | Present work |
|  | CPS119 | 2012 | MH031047 | Present work |
|  | CPS123a | 2012 | MH031052 | Present work |
|  | CPS124a | 2012 | MH031054 | Present work |
|  | CPS126a | 2012 | MH031058 | Present work |
|  | CPS127a | 2012 | MH031060 | Present work |
|  | CPS128 | 2012 | MH031062 | Present work |
|  | CPS130 | 2012 | MH031064 | Present work |
|  | CPS131a | 2012 | MH031065 | Present work |
|  | CPS133b | 2012 | MH031069 | Present work |
|  | CPS134 | 2012 | MH031070 | Present work |
|  | CPS135 | 2012 | MH031071 | Present work |
|  | CPS136a | 2012 | MH031072 | Present work |
|  | CPS137a | 2012 | MH031074 | Present work |
|  | CPS140 | 2012 | MH031080 | Present work |
|  | CPS142b | 2012 | MH031082 | Present work |
|  | CPS143b | 2012 | MH031084 | Present work |
|  | CPS144 | 2012 | MH031085 | Present work |
|  | CPS147 | 2012 | MH031088 | Present work |
|  | CPS148 | 2012 | MH031089 | Present work |
|  | CPS149a | 2012 | MH031090 | Present work |
|  | CPS151a | 2012 | MH031093 | Present work |
|  | CPS152a | 2012 | MH031095 | Present work |
|  | CPS153 | 2012 | MH031097 | Present work |
|  | CPS154a | 2012 | MH031098 | Present work |
|  | CPS155b | 2012 | MH031101 | Present work |
|  | CPS156b | 2012 | MH031103 | Present work |
|  | CPS158a | 2012 | MH031105 | Present work |
|  | CPS160a | 2012 | MH031108 | Present work |
|  | CPS162 | 2012 | MH031110 | Present work |
|  | CPS168a | 2012 | MH031118 | Present work |
|  | CPS170 | 2012 | MH031121 | Present work |
|  | CPS172a | 2012 | MH031123 | Present work |
|  | CPS173 | 2012 | MH031125 | Present work |
|  | CPS174 | 2012 | MH031126 | Present work |
|  | CPS175a | 2012 | MH031127 | Present work |
|  | CPS176a | 2012 | MH031129 | Present work |
|  | CPS177 | 2012 | MH031131 | Present work |
|  | CPN163a | 2014 | MH031139 | Present work |
|  | CPN165 | 2014 | MH031142 | Present work |
|  | CPN166a | 2014 | MH031143 | Present work |
|  | CPN168 | 2014 | MH031146 | Present work |
|  | CPN171 | 2014 | MH031147 | Present work |
|  | CPN174 | 2014 | MH031150 | Present work |
|  | CPN175 | 2014 | MH031151 | Present work |
|  | CPN176 | 2014 | MH031152 | Present work |
|  | CPN181a | 2014 | MH031158 | Present work |
|  | CPN182 | 2014 | MH031160 | Present work |
|  | CPN183a | 2014 | MH031161 | Present work |
|  | CPN186a | 2014 | MH031166 | Present work |
|  | CPN193a | 2014 | MH031174 | Present work |
|  | CPN196a | 2014 | MH031179 | Present work |
|  | CPN197a | 2014 | MH031181 | Present work |
|  | CPN199a | 2014 | MH031184 | Present work |
|  | CPN200 | 2014 | MH031186 | Present work |
|  | CPN201b | 2014 | MH031188 | Present work |
|  | CPN202 | 2014 | MH031189 | Present work |
|  | CPN206 | 2014 | MH031195 | Present work |
|  | CPN210 | 2014 | MH031199 | Present work |
|  | CPN212 | 2014 | MH031202 | Present work |
|  | CPN213 | 2014 | MH031203 | Present work |
|  | CPN214a | 2014 | MH031204 | Present work |
|  | CPN215a | 2014 | MH031206 | Present work |
|  | CPN216a | 2014 | MH031208 | Present work |
|  | CPN218a | 2014 | MH031212 | Present work |
|  | CPN219 | 2014 | MH031214 | Present work |
|  | CPN220a | 2014 | MH031215 | Present work |
|  | CPN221a | 2014 | MH031217 | Present work |
|  | CPN222a | 2014 | MH031219 | Present work |
|  | CPN224 | 2014 | MH031221 | Present work |
|  | CPN225 | 2014 | MH031222 | Present work |
|  | CPN226a | 2014 | MH031223 | Present work |
|  | CPN227 | 2014 | MH031225 | Present work |
|  | CPN230 | 2014 | MH031229 | Present work |
|  | CPN231 | 2014 | MH031230 | Present work |
|  | CPN233a | 2014 | MH031233 | Present work |
|  | CPN236 | 2014 | MH031238 | Present work |
|  | CPN237a | 2014 | MH031239 | Present work |
|  | CPN238a | 2014 | MH031241 | Present work |
|  | CPN240a | 2014 | MH031243 | Present work |
|  | CPN242 | 2014 | MH031247 | Present work |
|  | CPN243 | 2014 | MH031248 | Present work |
|  | CPN248 | 2014 | MH031251 | Present work |
|  | CPN250a | 2014 | MH031253 | Present work |
|  | CPN251 | 2014 | MH031255 | Present work |
|  | CPN253a | 2014 | MH031257 | Present work |
|  | CPN254 | 2014 | MH031259 | Present work |
|  | CPN255 | 2014 | MH031260 | Present work |
|  | CPN258 | 2014 | MH031264 | Present work |
| 2 | FT12b | 2005 | MH030890 | Present work |
|  | FT13b | 2005 | MH030892 | Present work |
|  | Lis5b | 2005 | MH030905 | Present work |
|  | Lis10b | 2005 | MH030911 | Present work |
|  | CPS124b | 2012 | MH031055 | Present work |
|  | CPN163b | 2014 | MH031140 | Present work |
|  | CPN197b | 2014 | MH031182 | Present work |
|  | CPN218b | 2014 | MH031213 | Present work |
| 3 | FT20a | 2005 | HM854968 | Francisco *et al*., 2008 |
|  | FT20b | 2005 | HM854969 | Francisco *et al*., 2008 |
|  | FT22a | 2005 | HM854970 | Francisco *et al*., 2008 |
|  | FT23a | 2005 | HM854972 | Francisco *et al*., 2008 |
|  | FT23b | 2005 | HM854973 | Francisco *et al*., 2008 |
| 4 | FT22b | 2005 | HM854971 | Francisco *et al*., 2008 |
| 5 | CPS26b | 2013 | MH030919 | Present work |
|  | CPS80b | 2013 | MH030989 | Present work |
|  | CPS137b | 2012 | MH031075 | Present work |
|  | CPS154b | 2012 | MH031099 | Present work |
|  | CPS176b | 2012 | MH031130 | Present work |
|  | CPN186b | 2014 | MH031167 | Present work |
|  | CPN214b | 2014 | MH031205 | Present work |
|  | CPN238b | 2014 | MH031242 | Present work |
|  | CPN250b | 2014 | MH031254 | Present work |
| 6 | CPS27 | 2013 | MH030920 | Present work |
| 7 | CPS29a | 2013 | MH030922 | Present work |
|  | CPS101a | 2012 | MH031018 | Present work |
|  | CPS163a | 2012 | MH031111 | Present work |
|  | CPS166a | 2012 | MH031115 | Present work |
|  | CPN179a | 2014 | MH031154 | Present work |
|  | CPN184a | 2014 | MH031163 | Present work |
|  | CPN190a | 2014 | MH031171 | Present work |
|  | CPN194a | 2014 | MH031176 | Present work |
|  | CPN234a | 2014 | MH031235 | Present work |
|  | CPN241a | 2014 | MH031245 | Present work |
|  | CPN245a | 2014 | MH031249 | Present work |
|  | CPN257a | 2014 | MH031262 | Present work |
| 8 | CPS29b | 2013 | MH030923 | Present work |
| 9 | CPS32 | 2013 | MH030926 | Present work |
| 10 | CPS33 | 2013 | MH030927 | Present work |
| 11 | CPS34 | 2013 | MH030928 | Present work |
| 12 | CPS36 | 2013 | MH030930 | Present work |
| 13 | CPS37b | 2013 | MH030932 | Present work |
| 14 | CPS39 | 2013 | MH030934 | Present work |
| 15 | CPS41a | 2013 | MH030936 | Present work |
| 16 | CPS41b | 2013 | MH030937 | Present work |
| 17 | CPS46 | 2013 | MH030942 | Present work |
| 18 | CPS47 | 2013 | MH030943 | Present work |
| 19 | CPS48 | 2013 | MH030944 | Present work |
| 20 | CPS49b | 2013 | MH030946 | Present work |
| 21 | CPS50b | 2013 | MH030948 | Present work |
| 22 | CPS51 | 2013 | MH030949 | Present work |
| 23 | CPS52a | 2013 | MH030950 | Present work |
| 24 | CPS52b | 2013 | MH030951 | Present work |
| 25 | CPS53 | 2013 | MH030952 | Present work |
| 26 | CPS54 | 2013 | MH030953 | Present work |
| 27 | CPS55 | 2013 | MH030954 | Present work |
| 28 | CPS57b | 2013 | MH030957 | Present work |
| 29 | CPS58a | 2013 | MH030958 | Present work |
| 30 | CPS58b | 2013 | MH030959 | Present work |
| 31 | CPS59a | 2013 | MH030960 | Present work |
| 32 | CPS60a | 2013 | MH030962 | Present work |
| 33 | CPS60b | 2013 | MH030963 | Present work |
| 34 | CPS61 | 2013 | MH030964 | Present work |
| 35 | CPS62b | 2013 | MH030966 | Present work |
| 36 | CPS63a | 2013 | MH030967 | Present work |
| 37 | CPS65 | 2013 | MH030969 | Present work |
| 38 | CPS66 | 2013 | MH030970 | Present work |
| 39 | CPS68b | 2013 | MH030973 | Present work |
| 40 | CPS72a | 2013 | MH030977 | Present work |
| 41 | CPS72b | 2013 | MH030978 | Present work |
| 42 | CPS74a | 2013 | MH030980 | Present work |
| 43 | CPS74b | 2013 | MH030981 | Present work |
| 44 | CPS75 | 2013 | MH030982 | Present work |
| 45 | CPS76 | 2013 | MH030983 | Present work |
| 46 | CPS77 | 2013 | MH030984 | Present work |
| 47 | CPS78a | 2013 | MH030985 | Present work |
| 48 | CPS79 | 2013 | MH030987 | Present work |
| 49 | CPS81a | 2013 | MH030990 | Present work |
| 50 | CPS81b | 2013 | MH030991 | Present work |
| 51 | CPS82b | 2013 | MH030993 | Present work |
| 52 | CPS83b | 2013 | MH030995 | Present work |
| 53 | CPS86b | 2013 | MH030999 | Present work |
| 54 | CPS88b | 2013 | MH031002 | Present work |
| 55 | CPS89 | 2012 | MH031003 | Present work |
| 56 | CPS90 | 2012 | MH031004 | Present work |
| 57 | CPS91 | 2012 | MH031005 | Present work |
| 58 | CPS92b | 2012 | MH031007 | Present work |
| 59 | CPS93 | 2012 | MH031008 | Present work |
| 60 | CPS94 | 2012 | MH031009 | Present work |
| 61 | CPS96 | 2012 | MH031011 | Present work |
| 62 | CPS98b | 2012 | MH031014 | Present work |
| 63 | CPS100b | 2012 | MH031017 | Present work |
| 64 | CPS101b | 2012 | MH031019 | Present work |
| 65 | CPS102a | 2012 | MH031020 | Present work |
| 66 | CPS102b | 2012 | MH031021 | Present work |
| 67 | CPS103b | 2012 | MH031023 | Present work |
| 68 | CPS104b | 2012 | MH031025 | Present work |
| 69 | CPS105b | 2012 | MH031027 | Present work |
| 70 | CPS106b | 2012 | MH031029 | Present work |
| 71 | CPS107 | 2012 | MH031030 | Present work |
| 72 | CPS108 | 2012 | MH031031 | Present work |
| 73 | CPS109 | 2012 | MH031032 | Present work |
| 74 | CPS110a | 2012 | MH031033 | Present work |
| 75 | CPS110b | 2012 | MH031034 | Present work |
| 76 | CPS111b | 2012 | MH031036 | Present work |
| 77 | CPS116b | 2012 | MH031042 | Present work |
| 78 | CPS117b | 2012 | MH031044 | Present work |
| 79 | CPS118a | 2012 | MH031045 | Present work |
| 80 | CPS118b | 2012 | MH031046 | Present work |
| 81 | CPS121a | 2012 | MH031048 | Present work |
| 82 | CPS121b | 2012 | MH031049 | Present work |
| 83 | CPS122a | 2012 | MH031050 | Present work |
| 84 | CPS122b | 2012 | MH031051 | Present work |
| 85 | CPS123b | 2012 | MH031053 | Present work |
| 86 | CPS125a | 2012 | MH031056 | Present work |
| 87 | CPS125b | 2012 | MH031057 | Present work |
| 88 | CPS126b | 2012 | MH031059 | Present work |
| 89 | CPS127b | 2012 | MH031061 | Present work |
| 90 | CPS129 | 2012 | MH031063 | Present work |
| 91 | CPS131b | 2012 | MH031066 | Present work |
| 92 | CPS132 | 2012 | MH031067 | Present work |
| 93 | CPS133a | 2012 | MH031068 | Present work |
| 94 | CPS136b | 2012 | MH031073 | Present work |
| 95 | CPS138a | 2012 | MH031076 | Present work |
| 96 | CPS138b | 2012 | MH031077 | Present work |
| 97 | CPS139a | 2012 | MH031078 | Present work |
| 98 | CPS139b | 2012 | MH031079 | Present work |
| 99 | CPS142a | 2012 | MH031081 | Present work |
| 100 | CPS143a | 2012 | MH031083 | Present work |
| 101 | CPS145 | 2012 | MH031086 | Present work |
| 102 | CPS146 | 2012 | MH031087 | Present work |
| 103 | CPS149b | 2012 | MH031091 | Present work |
| 104 | CPS150 | 2012 | MH031092 | Present work |
| 105 | CPS151b | 2012 | MH031094 | Present work |
| 106 | CPS152b | 2012 | MH031096 | Present work |
| 107 | CPS155a | 2012 | MH031100 | Present work |
| 108 | CPS156a | 2012 | MH031102 | Present work |
| 109 | CPS157 | 2012 | MH031104 | Present work |
| 110 | CPS158b | 2012 | MH031106 | Present work |
| 111 | CPS159 | 2012 | MH031107 | Present work |
| 112 | CPS160b | 2012 | MH031109 | Present work |
| 113 | CPS163b | 2012 | MH031112 | Present work |
| 114 | CPS165a | 2012 | MH031113 | Present work |
| 115 | CPS165b | 2012 | MH031114 | Present work |
| 116 | CPS166b | 2012 | MH031116 | Present work |
| 117 | CPS167 | 2012 | MH031117 | Present work |
| 118 | CPS168b | 2012 | MH031119 | Present work |
| 119 | CPS169 | 2012 | MH031120 | Present work |
| 120 | CPS171 | 2012 | MH031122 | Present work |
| 121 | CPS172b | 2012 | MH031124 | Present work |
| 122 | CPS175b | 2012 | MH031128 | Present work |
| 123 | CPS178a | 2012 | MH031132 | Present work |
| 124 | CPS178b | 2012 | MH031133 | Present work |
| 125 | CPS179 | 2012 | MH031134 | Present work |
| 126 | CPS180a | 2012 | MH031135 | Present work |
| 127 | CPS180b | 2012 | MH031136 | Present work |
| 128 | CPN161 | 2014 | MH031137 | Present work |
| 129 | CPN162 | 2014 | MH031138 | Present work |
| 130 | CPN164 | 2014 | MH031141 | Present work |
| 131 | CPN166b | 2014 | MH031144 | Present work |
| 132 | CPN167 | 2014 | MH031145 | Present work |
| 133 | CPN172 | 2014 | MH031148 | Present work |
| 134 | CPN173 | 2014 | MH031149 | Present work |
| 135 | CPN177 | 2014 | MH031153 | Present work |
| 136 | CPN179b | 2014 | MH031155 | Present work |
| 137 | CPN180a | 2014 | MH031156 | Present work |
| 138 | CPN180b | 2014 | MH031157 | Present work |
| 139 | CPN181b | 2014 | MH031159 | Present work |
| 140 | CPN183b | 2014 | MH031162 | Present work |
| 141 | CPN184b | 2014 | MH031164 | Present work |
| 142 | CPN185 | 2014 | MH031165 | Present work |
| 143 | CPN187 | 2014 | MH031168 | Present work |
| 144 | CPN188 | 2014 | MH031169 | Present work |
| 145 | CPN189 | 2014 | MH031170 | Present work |
| 146 | CPN190b | 2014 | MH031172 | Present work |
| 147 | CPN192 | 2014 | MH031173 | Present work |
| 148 | CPN193b | 2014 | MH031175 | Present work |
| 149 | CPN194b | 2014 | MH031177 | Present work |
| 150 | CPN195 | 2014 | MH031178 | Present work |
| 151 | CPN196b | 2014 | MH031180 | Present work |
| 152 | CPN198 | 2014 | MH031183 | Present work |
| 153 | CPN199b | 2014 | MH031185 | Present work |
| 154 | CPN201a | 2014 | MH031187 | Present work |
| 155 | CPN203 | 2014 | MH031190 | Present work |
| 156 | CPN204a | 2014 | MH031191 | Present work |
| 157 | CPN204b | 2014 | MH031192 | Present work |
| 158 | CPN205a | 2014 | MH031193 | Present work |
| 159 | CPN205b | 2014 | MH031194 | Present work |
| 160 | CPN207 | 2014 | MH031196 | Present work |
| 161 | CPN208 | 2014 | MH031197 | Present work |
| 162 | CPN209 | 2014 | MH031198 | Present work |
| 163 | CPN211a | 2014 | MH031200 | Present work |
| 164 | CPN211b | 2014 | MH031201 | Present work |
| 165 | CPN215b | 2014 | MH031207 | Present work |
| 166 | CPN216b | 2014 | MH031209 | Present work |
| 167 | CPN217a | 2014 | MH031210 | Present work |
| 168 | CPN217b | 2014 | MH031211 | Present work |
| 169 | CPN220b | 2014 | MH031216 | Present work |
| 170 | CPN221b | 2014 | MH031218 | Present work |
| 171 | CPN222b | 2014 | MH031220 | Present work |
| 172 | CPN226b | 2014 | MH031224 | Present work |
| 173 | CPN228a | 2014 | MH031226 | Present work |
| 174 | CPN228b | 2014 | MH031227 | Present work |
| 175 | CPN229 | 2014 | MH031228 | Present work |
| 176 | CPN232a | 2014 | MH031231 | Present work |
| 177 | CPN232b | 2014 | MH031232 | Present work |
| 178 | CPN233b | 2014 | MH031234 | Present work |
| 179 | CPN234b | 2014 | MH031236 | Present work |
| 180 | CPN235 | 2014 | MH031237 | Present work |
| 181 | CPN237b | 2014 | MH031240 | Present work |
| 182 | CPN240b | 2014 | MH031244 | Present work |
| 183 | CPN241b | 2014 | MH031246 | Present work |
| 184 | CPN245b | 2014 | MH031250 | Present work |
| 185 | CPN249 | 2014 | MH031252 | Present work |
| 186 | CPN252 | 2014 | MH031256 | Present work |
| 187 | CPN253b | 2014 | MH031258 | Present work |
| 188 | CPN256 | 2014 | MH031261 | Present work |
| 189 | CPN257b | 2014 | MH031263 | Present work |
| 190 | CPN259a | 2014 | MH031265 | Present work |
| 191 | CPN259b | 2014 | MH031266 | Present work |
| 192 | CPN260 | 2014 | MH031267 | Present work |
| 193 | CPN263a | 2014 | MH031268 | Present work |
| 194 | CPN263b | 2014 | MH031269 | Present work |
| 195 | CPN265 | 2014 | MH031270 | Present work |
| 196 | CPS64a | 2013 | MH031271 | Present work |
| 197 | CPS64b | 2013 | MH031272 | Present work |
|  |  |  |  |  |

Table S3 – Sampling periods, specimens and Genebank Accession Numbers for CR and S7 of *Lipophrys pholis*.

|  |  |  |  |  |  |
| --- | --- | --- | --- | --- | --- |
| Genetic Marker | Haplotype number | Specimen | Sampling year | Accession number | Work |
| *CR* | 1 | CPN211 | 2014 | KR028917 | Francisco & Robalo, 2015 |
|  |  | CPN261 | 2014 | KR028961 | Francisco & Robalo, 2015 |
|  |  | SP36 | 2003 | DQ154185 | Francisco et al., 2006b |
|  |  | SP38 | 2003 | DQ154186 | Francisco et al., 2006b |
|  |  | CPN106 | 2013 | KR028827 | Francisco & Robalo, 2015 |
|  |  | CPN72 | 2013 | KR028798 | Francisco & Robalo, 2015 |
|  | 2 | CPN85 | 2013 | KR028809 | Francisco & Robalo, 2015 |
|  | 3 | CPN136 | 2013 | KR028853 | Francisco & Robalo, 2015 |
|  | 4 | CPN273 | 2014 | KR028972 | Francisco & Robalo, 2015 |
|  | 5 | CPN213 | 2014 | KR028919 | Francisco & Robalo, 2015 |
|  | 6 | CPN223 | 2014 | KR028928 | Francisco & Robalo, 2015 |
|  | 7 | CPN254 | 2014 | KR028956 | Francisco & Robalo, 2015 |
|  | 8 | CPN209 | 2014 | KR028915 | Francisco & Robalo, 2015 |
|  | 9 | CPN73 | 2013 | KR028799 | Francisco & Robalo, 2015 |
|  | 10 | CPN242 | 2014 | KR028947 | Francisco & Robalo, 2015 |
|  | 11 | SP35 | 2003 | DQ154192 | Francisco et al., 2006b |
|  | 12 | CPN262 | 2014 | KR028962 | Francisco & Robalo, 2015 |
|  | 13 | CPN204 | 2014 | KR028910 | Francisco & Robalo, 2015 |
|  | 14 | CPN243 | 2014 | KR028948 | Francisco & Robalo, 2015 |
|  |  | CPN114 | 2013 | KR028835 | Francisco & Robalo, 2015 |
|  |  | CPN171 | 2013 | KR028882 | Francisco & Robalo, 2015 |
|  | 15 | CPN83 | 2013 | KR028807 | Francisco & Robalo, 2015 |
|  | 16 | CPN120 | 2013 | KR028839 | Francisco & Robalo, 2015 |
|  | 17 | CPN238 | 2014 | KR028943 | Francisco & Robalo, 2015 |
|  | 18 | CPN216 | 2014 | KR028921 | Francisco & Robalo, 2015 |
|  | 19 | CPN215 | 2014 | KR028920 | Francisco & Robalo, 2015 |
|  | 20 | SP57 | 2003 | DQ154199 | Francisco et al., 2006b |
|  | 21 | SP56 | 2003 | DQ154211 | Francisco et al., 2006b |
|  |  | CPN172 | 2013 | KR028883 | Francisco & Robalo, 2015 |
|  | 22 | CPN123 | 2013 | KR028841 | Francisco & Robalo, 2015 |
|  | 23 | CPN63 | 2013 | KR028789 | Francisco & Robalo, 2015 |
|  | 24 | CPN284 | 2014 | KR028980 | Francisco & Robalo, 2015 |
|  | 25 | CPN277 | 2014 | KR028976 | Francisco & Robalo, 2015 |
|  | 26 | CPN285 | 2014 | KR028981 | Francisco & Robalo, 2015 |
|  | 27 | CPN276 | 2014 | KR028975 | Francisco & Robalo, 2015 |
|  | 28 | CPN109 | 2013 | KR028830 | Francisco & Robalo, 2015 |
|  |  | CPN173 | 2013 | KR028884 | Francisco & Robalo, 2015 |
|  |  | CPN95 | 2013 | KR028816 | Francisco & Robalo, 2015 |
|  | 29 | CPN267 | 2014 | KR028966 | Francisco & Robalo, 2015 |
|  | 30 | CPN283 | 2014 | KR028979 | Francisco & Robalo, 2015 |
|  | 31 | SP21 | 2003 | DQ154236 | Francisco et al., 2006b |
|  |  | SP33 | 2003 | DQ154237 | Francisco et al., 2006b |
|  | 32 | CPN143 | 2013 | KR028860 | Francisco & Robalo, 2015 |
|  | 33 | CPN144 | 2013 | KR028861 | Francisco & Robalo, 2015 |
|  | 34 | CPN234 | 2014 | KR028939 | Francisco & Robalo, 2015 |
|  |  | CPN233 | 2014 | KR028938 | Francisco & Robalo, 2015 |
|  | 35 | CPN90 | 2013 | KR028812 | Francisco & Robalo, 2015 |
|  | 36 | CPN107 | 2013 | KR028828 | Francisco & Robalo, 2015 |
|  |  | CPN149 | 2013 | KR028866 | Francisco & Robalo, 2015 |
|  | 37 | CPN133 | 2013 | KR028850 | Francisco & Robalo, 2015 |
|  | 38 | CPN165 | 2013 | KR028630 | Francisco & Robalo, 2015 |
|  | 39 | CPN152 | 2013 | KR028869 | Francisco & Robalo, 2015 |
|  | 40 | SP26 | 2003 | DQ154194 | Francisco et al., 2006b |
|  |  | CPN147 | 2013 | KR028864 | Francisco & Robalo, 2015 |
|  | 41 | CPN239 | 2014 | KR028944 | Francisco & Robalo, 2015 |
|  | 42 | CPN189 | 2014 | KR028896 | Francisco & Robalo, 2015 |
|  | 43 | CPN268 | 2014 | KR028967 | Francisco & Robalo, 2015 |
|  |  | SP6 | 2003 | DQ154197 | Francisco et al., 2006b |
|  |  | CPN75 | 2013 | KR028801 | Francisco & Robalo, 2015 |
|  | 44 | CPN255 | 2014 | KR028957 | Francisco & Robalo, 2015 |
|  | 45 | CPN177 | 2014 | KR028887 | Francisco & Robalo, 2015 |
|  | 46 | CPN197 | 2013 | KR028903 | Francisco & Robalo, 2015 |
|  |  | CPN122 | 2013 | KR028840 | Francisco & Robalo, 2015 |
|  |  | CPN68 | 2013 | KR028794 | Francisco & Robalo, 2015 |
|  | 47 | CPN137 | 2013 | KR028854 | Francisco & Robalo, 2015 |
|  | 48 | SP8 | 2003 | DQ154191 | Francisco et al., 2006b |
|  |  | CPN116 | 2013 | KR028836 | Francisco & Robalo, 2015 |
|  | 49 | CPN222 | 2014 | KR028927 | Francisco & Robalo, 2015 |
|  | 50 | CPN113 | 2013 | KR028834 | Francisco & Robalo, 2015 |
|  | 51 | CPN110 | 2013 | KR028831 | Francisco & Robalo, 2015 |
|  | 52 | CPN142 | 2013 | KR028859 | Francisco & Robalo, 2015 |
|  | 53 | CPN151 | 2013 | KR028868 | Francisco & Robalo, 2015 |
|  | 54 | CPN193 | 2014 | KR028899 | Francisco & Robalo, 2015 |
|  | 55 | CPN212 | 2014 | KR028918 | Francisco & Robalo, 2015 |
|  | 56 | CPN286 | 2014 | KR028982 | Francisco & Robalo, 2015 |
|  |  | CPN180 | 2014 | KR028889 | Francisco & Robalo, 2015 |
|  | 57 | CPN96 | 2013 | KR028817 | Francisco & Robalo, 2015 |
|  | 58 | CPN231 | 2014 | KR028936 | Francisco & Robalo, 2015 |
|  | 59 | CPN220 | 2014 | KR028925 | Francisco & Robalo, 2015 |
|  | 60 | CPN240 | 2014 | KR028945 | Francisco & Robalo, 2015 |
|  | 61 | CPN264 | 2014 | KR028964 | Francisco & Robalo, 2015 |
|  |  | CPN237 | 2014 | KR028942 | Francisco & Robalo, 2015 |
|  | 62 | CPN169 | 2013 | KR028880 | Francisco & Robalo, 2015 |
|  | 63 | CPN201 | 2014 | KR028907 | Francisco & Robalo, 2015 |
|  | 64 | SP29 | 2003 | DQ154187 | Francisco et al., 2006b |
|  | 65 | CPN269 | 2014 | KR028968 | Francisco & Robalo, 2015 |
|  | 66 | CPN198 | 2014 | KR028904 | Francisco & Robalo, 2015 |
|  | 67 | SP53 | 2003 | DQ154189 | Francisco et al., 2006b |
|  | 68 | CPN208 | 2014 | KR028914 | Francisco & Robalo, 2015 |
|  | 69 | CPN130 | 2013 | KR028846 | Francisco & Robalo, 2015 |
|  | 70 | SP31 | 2003 | DQ154209 | Francisco et al., 2006b |
|  | 71 | CPN265 | 2014 | KR028965 | Francisco & Robalo, 2015 |
|  | 72 | CPN187 | 2014 | KR028894 | Francisco & Robalo, 2015 |
|  | 73 | SP13 | 2003 | DQ154213 | Francisco et al., 2006b |
|  | 74 | CPN218 | 2014 | KR028923 | Francisco & Robalo, 2015 |
|  | 75 | CPN111 | 2013 | KR028832 | Francisco & Robalo, 2015 |
|  | 76 | CPN250 | 2014 | KR028953 | Francisco & Robalo, 2015 |
|  | 77 | CPN225 | 2014 | KR028930 | Francisco & Robalo, 2015 |
|  | 78 | CPN282 | 2014 | KR028978 | Francisco & Robalo, 2015 |
|  | 79 | CPN191 | 2014 | KR028897 | Francisco & Robalo, 2015 |
|  | 80 | CPN271 | 2014 | KR028970 | Francisco & Robalo, 2015 |
|  | 81 | CPN241 | 2014 | KR028946 | Francisco & Robalo, 2015 |
|  |  | SP55 | 2003 | DQ154227 | Francisco et al., 2006b |
|  | 82 | CPN183 | 2014 | KR028890 | Francisco & Robalo, 2015 |
|  |  | CPN219 | 2014 | KR028924 | Francisco & Robalo, 2015 |
|  |  | SP12 | 2003 | DQ154224 | Francisco et al., 2006b |
|  |  | SP48 | 2003 | DQ154225 | Francisco et al., 2006b |
|  | 83 | CPN230 | 2014 | KR028935 | Francisco & Robalo, 2015 |
|  | 84 | CPN259 | 2014 | KR028959 | Francisco & Robalo, 2015 |
|  | 85 | CPN260 | 2014 | KR028960 | Francisco & Robalo, 2015 |
|  | 86 | CPN97 | 2013 | KR028818 | Francisco & Robalo, 2015 |
|  | 87 | CPN105 | 2013 | KR028826 | Francisco & Robalo, 2015 |
|  |  | CPN129 | 2013 | KR028845 | Francisco & Robalo, 2015 |
|  |  | CPN64 | 2013 | KR028790 | Francisco & Robalo, 2015 |
|  |  | CPN65 | 2013 | KR028791 | Francisco & Robalo, 2015 |
|  |  | CPN93 | 2013 | KR028815 | Francisco & Robalo, 2015 |
|  |  | CPN98 | 2013 | KR028819 | Francisco & Robalo, 2015 |
|  |  | CPN99 | 2013 | KR028820 | Francisco & Robalo, 2015 |
|  |  | CPN158 | 2013 | KR028872 | Francisco & Robalo, 2015 |
|  | 88 | CPN104 | 2013 | KR028825 | Francisco & Robalo, 2015 |
|  | 89 | CPN70 | 2013 | KR028796 | Francisco & Robalo, 2015 |
|  | 90 | CPN76 | 2013 | KR028802 | Francisco & Robalo, 2015 |
|  |  | CPN79 | 2013 | KR028805 | Francisco & Robalo, 2015 |
|  | 91 | CPN146 | 2013 | KR028863 | Francisco & Robalo, 2015 |
|  | 92 | CPN166 | 2013 | KR028876 | Francisco & Robalo, 2015 |
|  | 93 | CPN71 | 2013 | KR028797 | Francisco & Robalo, 2015 |
|  | 94 | CPN192 | 2014 | KR028898 | Francisco & Robalo, 2015 |
|  | 95 | SP2 | 2003 | DQ154214 | Francisco et al., 2006b |
|  | 96 | CPN235 | 2014 | KR028940 | Francisco & Robalo, 2015 |
|  | 97 | SP47 | 2003 | DQ154231 | Francisco et al., 2006b |
|  | 98 | CPN185 | 2014 | KR028892 | Francisco & Robalo, 2015 |
|  | 99 | CPN232 | 2014 | KR028937 | Francisco & Robalo, 2015 |
|  | 100 | CPN206 | 2014 | KR028912 | Francisco & Robalo, 2015 |
|  |  | CPN199 | 2014 | KR028905 | Francisco & Robalo, 2015 |
|  |  | CPN217 | 2014 | KR028922 | Francisco & Robalo, 2015 |
|  | 101 | SP7 | 2003 | DQ154222 | Francisco et al., 2006b |
|  | 102 | CPN69 | 2013 | KR028795 | Francisco & Robalo, 2015 |
|  |  | CPN77 | 2013 | KR028803 | Francisco & Robalo, 2015 |
|  | 103 | CPN100 | 2013 | KR028821 | Francisco & Robalo, 2015 |
|  | 104 | CPN101 | 2013 | KR028822 | Francisco & Robalo, 2015 |
|  | 105 | CPN117 | 2013 | KR028837 | Francisco & Robalo, 2015 |
|  | 106 | CPN81 | 2013 | KR028806 | Francisco & Robalo, 2015 |
|  | 107 | CPN92 | 2013 | KR028814 | Francisco & Robalo, 2015 |
|  | 108 | CPN195 | 2014 | KR028901 | Francisco & Robalo, 2015 |
|  | 109 | CPN188 | 2014 | KR028895 | Francisco & Robalo, 2015 |
|  | 110 | CPN178 | 2014 | KR028888 | Francisco & Robalo, 2015 |
|  | 111 | CPN247 | 2014 | KR028951 | Francisco & Robalo, 2015 |
|  | 112 | CPN200 | 2014 | KR028906 | Francisco & Robalo, 2015 |
|  | 113 | CPN249 | 2014 | KR028952 | Francisco & Robalo, 2015 |
|  | 114 | CPN207 | 2014 | KR028913 | Francisco & Robalo, 2015 |
|  | 115 | CPN67 | 2013 | KR028793 | Francisco & Robalo, 2015 |
|  | 116 | CPN1321 | 2013 | KR028848 | Francisco & Robalo, 2015 |
|  | 117 | CPN132 | 2013 | KR028849 | Francisco & Robalo, 2015 |
|  | 118 | CPN74 | 2013 | KR028800 | Francisco & Robalo, 2015 |
|  |  | CPN91 | 2013 | KR028813 | Francisco & Robalo, 2015 |
|  | 119 | CPN156 | 2013 | KR028871 | Francisco & Robalo, 2015 |
|  | 120 | CPN84 | 2013 | KR028808 | Francisco & Robalo, 2015 |
|  | 121 | CPN140 | 2013 | KR028857 | Francisco & Robalo, 2015 |
|  |  | CPN141 | 2013 | KR028858 | Francisco & Robalo, 2015 |
|  | 122 | CPN160 | 2013 | KR028873 | Francisco & Robalo, 2015 |
|  | 123 | CPN118 | 2013 | KR028838 | Francisco & Robalo, 2015 |
|  | 124 | CPN86 | 2013 | KR028810 | Francisco & Robalo, 2015 |
|  | 125 | CPN153 | 2013 | KR028870 | Francisco & Robalo, 2015 |
|  |  | CPN162 | 2013 | KR028874 | Francisco & Robalo, 2015 |
|  | 126 | CPN174 | 2013 | KR028885 | Francisco & Robalo, 2015 |
|  | 127 | CPN148 | 2013 | KR028865 | Francisco & Robalo, 2015 |
|  | 128 | CPN205 | 2014 | KR028911 | Francisco & Robalo, 2015 |
|  | 129 | CPN138 | 2014 | KR028855 | Francisco & Robalo, 2015 |
|  | 130 | SP52 | 2003 | DQ154183 | Francisco et al., 2006b |
|  | 131 | CPN203 | 2014 | KR028909 | Francisco & Robalo, 2015 |
|  | 132 | CPN274 | 2014 | KR028973 | Francisco & Robalo, 2015 |
|  | 133 | SP54 | 2003 | DQ154246 | Francisco et al., 2006b |
|  | 134 | CPN228 | 2014 | KR028933 | Francisco & Robalo, 2015 |
|  | 135 | SP23 | 2003 | DQ154244 | Francisco et al., 2006b |
|  | 136 | CPN202 | 2014 | KR028907 | Francisco & Robalo, 2015 |
|  | 137 | CPN102 | 2013 | KR028823 | Francisco & Robalo, 2015 |
|  | 138 | CPN229 | 2014 | KR028934 | Francisco & Robalo, 2015 |
|  | 139 | CPN145 | 2013 | KR028862 | Francisco & Robalo, 2015 |
|  | 140 | SP46 | 2003 | DQ154255 | Francisco et al., 2006b |
|  |  | CPN103 | 2013 | KR028824 | Francisco & Robalo, 2015 |
|  | 141 | CPN224 | 2014 | KR028929 | Francisco & Robalo, 2015 |
|  | 142 | CPN252 | 2014 | KR028954 | Francisco & Robalo, 2015 |
|  |  | CPN253 | 2014 | KR028955 | Francisco & Robalo, 2015 |
|  | 143 | SP24 | 2003 | DQ154250 | Francisco et al., 2006b |
|  | 144 | CPN1312 | 2013 | KR028847 | Francisco & Robalo, 2015 |
|  | 145 | CPN210 | 2014 | KR028916 | Francisco & Robalo, 2015 |
|  |  | CPN186 | 2014 | KR028893 | Francisco & Robalo, 2015 |
|  | 146 | CPN288 | 2014 | KR028984 | Francisco & Robalo, 2015 |
|  | 147 | CPN287 | 2014 | KR028983 | Francisco & Robalo, 2015 |
|  | 148 | CPN176 | 2014 | KR028886 | Francisco & Robalo, 2015 |
|  | 149 | CPN168 | 2013 | KR028879 | Francisco & Robalo, 2015 |
|  | 150 | CPN134 | 2013 | KR028851 | Francisco & Robalo, 2015 |
|  | 151 | CPN226 | 2014 | KR028931 | Francisco & Robalo, 2015 |
|  | 152 | CPN139 | 2013 | KR028856 | Francisco & Robalo, 2015 |
|  | 153 | CPN270 | 2014 | KR028969 | Francisco & Robalo, 2015 |
|  | 154 | CPN167 | 2013 | KR028878 | Francisco & Robalo, 2015 |
|  | 155 | CPN135 | 2013 | KR028852 | Francisco & Robalo, 2015 |
|  | 156 | CPN246 | 2014 | KR028950 | Francisco & Robalo, 2015 |
|  | 157 | CPN263 | 2014 | KR028963 | Francisco & Robalo, 2015 |
|  | 158 | CPN194 | 2014 | KR028900 | Francisco & Robalo, 2015 |
|  | 159 | CPN279 | 2014 | KR028977 | Francisco & Robalo, 2015 |
|  |  | SP37 | 2003 | DQ154177 | Francisco et al., 2006b |
|  |  | CPN108 | 2013 | KR028829 | Francisco & Robalo, 2015 |
|  |  | CPN112 | 2013 | KR028833 | Francisco & Robalo, 2015 |
|  |  | CPN124 | 2013 | KR028842 | Francisco & Robalo, 2015 |
|  |  | CPN126 | 2013 | KR028844 | Francisco & Robalo, 2015 |
|  |  | CPN66 | 2013 | KR028792 | Francisco & Robalo, 2015 |
|  |  | CPN78 | 2013 | KR028804 | Francisco & Robalo, 2015 |
|  | 160 | CPN184 | 2014 | KR028891 | Francisco & Robalo, 2015 |
|  | 161 | CPN236 | 2014 | KR028941 | Francisco & Robalo, 2015 |
|  | 162 | CPN227 | 2014 | KR028932 | Francisco & Robalo, 2015 |
|  | 163 | CPN272 | 2014 | KR028971 | Francisco & Robalo, 2015 |
|  |  | CPN257 | 2014 | KR028958 | Francisco & Robalo, 2015 |
|  |  | SP50 | 2003 | DQ154171 | Francisco et al., 2006b |
|  |  | SP51 | 2003 | DQ154172 | Francisco et al., 2006b |
|  |  | CPN125 | 2013 | KR028843 | Francisco & Robalo, 2015 |
|  |  | CPN89 | 2013 | KR028811 | Francisco & Robalo, 2015 |
|  | 164 | CPN150 | 2013 | KR028867 | Francisco & Robalo, 2015 |
|  | 165 | CPN275 | 2014 | KR028974 | Francisco & Robalo, 2015 |
|  | 166 | CPN196 | 2014 | KR028902 | Francisco & Robalo, 2015 |
|  | 167 | CPN244 | 2014 | KR028949 | Francisco & Robalo, 2015 |
|  |  | CPN221 | 2014 | KR028926 | Francisco & Robalo, 2015 |
|  | 168 | CPN170 | 2013 | KR028881 | Francisco & Robalo, 2015 |
|  | 169 | CPN164 | 2013 | KR028875 | Francisco & Robalo, 2015 |
|  | 170 | SP14 | 2003 | DQ154182 | Francisco et al., 2006b |
|  | 171 | SP17 | 2003 | DQ154249 | Francisco et al., 2006b |
| S7 | 1 | SP35a | 2003 | GU302140 | Francisco et al., 2011 |
|  |  | SP35b | 2003 | GU302141 | Francisco et al., 2011 |
|  |  | SP37a | 2003 | GU301992 | Francisco et al., 2011 |
|  |  | SP37b | 2003 | GU302142 | Francisco et al., 2011 |
|  |  | SP38a | 2003 | GU302143 | Francisco et al., 2011 |
|  |  | SP39a | 2003 | GU302144 | Francisco et al., 2011 |
|  |  | SP40b | 2003 | GU302145 | Francisco et al., 2011 |
|  |  | SP42b | 2003 | GU302146 | Francisco et al., 2011 |
|  |  | SP45b | 2003 | GU302147 | Francisco et al., 2011 |
|  |  | SP48a | 2003 | GU302148 | Francisco et al., 2011 |
|  |  | SP48b | 2003 | GU302149 | Francisco et al., 2011 |
|  |  | SP49b | 2003 | GU302150 | Francisco et al., 2011 |
|  |  | SP51b | 2003 | GU302151 | Francisco et al., 2011 |
|  |  | SP52a | 2003 | GU302152 | Francisco et al., 2011 |
|  |  | SP52b | 2003 | GU302153 | Francisco et al., 2011 |
|  |  | SP53a | 2003 | GU302154 | Francisco et al., 2011 |
|  |  | SP53b | 2003 | GU302155 | Francisco et al., 2011 |
|  |  | SP54b | 2003 | GU302156 | Francisco et al., 2011 |
|  |  | SP55b | 2003 | GU302157 | Francisco et al., 2011 |
|  |  | SP56b | 2003 | GU302158 | Francisco et al., 2011 |
|  |  | SP57b | 2003 | GU302159 | Francisco et al., 2011 |
|  |  | CPN63 | 2013 | MH024090 | present work |
|  |  | CPN69a | 2013 | MH024095 | present work |
|  |  | CPN80a | 2013 | MH024101 | present work |
|  |  | CPN83 | 2013 | MH024105 | present work |
|  |  | CPN86 | 2013 | MH024110 | present work |
|  |  | CPN103 | 2013 | MH024136 | present work |
|  |  | CPN114 | 2013 | MH024153 | present work |
|  |  | CPN116b | 2013 | MH024157 | present work |
|  |  | CPN122 | 2013 | MH024162 | present work |
|  |  | CPN123 | 2013 | MH024163 | present work |
|  |  | CPN125 | 2013 | MH024166 | present work |
|  |  | CPN137 | 2013 | MH024179 | present work |
|  |  | CPN139 | 2013 | MH024182 | present work |
|  |  | CPN140 | 2013 | MH024183 | present work |
|  |  | CPN142 | 2013 | MH024186 | present work |
|  |  | CPN143b | 2013 | MH024188 | present work |
|  |  | CPN151 | 2013 | MH024202 | present work |
|  |  | CPN152 | 2013 | MH024203 | present work |
|  |  | CPN153 | 2013 | MH024204 | present work |
|  |  | CPN159 | 2013 | MH024211 | present work |
|  |  | CPN162 | 2013 | MH024214 | present work |
|  |  | CPN164 | 2013 | MH024215 | present work |
|  |  | CPN165 | 2013 | MH024216 | present work |
|  |  | CPN168b | 2013 | MH024220 | present work |
|  |  | CPN179 | 2014 | MH024223 | present work |
|  |  | CPN182 | 2014 | MH024224 | present work |
|  |  | CPN186 | 2014 | MH024229 | present work |
|  |  | CPN190 | 2014 | MH024234 | present work |
|  |  | CPN192 | 2014 | MH024237 | present work |
|  |  | CPN196a | 2014 | MH024244 | present work |
|  |  | CPN199 | 2014 | MH024248 | present work |
|  |  | CPN201 | 2014 | MH024251 | present work |
|  |  | CPN202 | 2014 | MH024252 | present work |
|  |  | CPN205 | 2014 | MH024257 | present work |
|  |  | CPN209 | 2014 | MH024264 | present work |
|  |  | CPN212 | 2014 | MH024267 | present work |
|  |  | CPN214 | 2014 | MH024270 | present work |
|  |  | CPN218b | 2014 | MH024274 | present work |
|  |  | CPN219a | 2014 | MH024275 | present work |
|  |  | CPN224 | 2014 | MH024283 | present work |
|  |  | CPN225 | 2014 | MH024284 | present work |
|  |  | CPN230 | 2014 | MH024288 | present work |
|  |  | CPN231b | 2014 | MH024290 | present work |
|  |  | CPN235 | 2014 | MH024295 | present work |
|  |  | CPN238 | 2014 | MH024300 | present work |
|  |  | CPN239 | 2014 | MH024301 | present work |
|  |  | CPN241 | 2014 | MH024304 | present work |
|  |  | CPN244b | 2014 | MH024310 | present work |
|  |  | CPN246a | 2014 | MH024313 | present work |
|  |  | CPN249 | 2014 | MH024317 | present work |
|  |  | CPN250b | 2014 | MH024319 | present work |
|  |  | CPN251 | 2014 | MH024320 | present work |
|  |  | CPN254 | 2014 | MH024325 | present work |
|  |  | CPN255 | 2014 | MH024326 | present work |
|  |  | CPN264b | 2014 | MH024338 | present work |
|  |  | CPN266b | 2014 | MH024342 | present work |
|  |  | CPN269 | 2014 | MH024345 | present work |
|  |  | CPN271 | 2014 | MH024348 | present work |
|  |  | CPN272 | 2014 | MH024349 | present work |
|  |  | CPN275 | 2014 | MH024354 | present work |
|  |  | CPN276 | 2014 | MH024355 | present work |
|  | 2 | SP41a | 2003 | GU301993 | Francisco et al., 2011 |
|  |  | SP42a | 2003 | GU301994 | Francisco et al., 2011 |
|  |  | SP45a | 2003 | GU301995 | Francisco et al., 2011 |
|  |  | SP46a | 2003 | GU301996 | Francisco et al., 2011 |
|  |  | SP46b | 2003 | GU301997 | Francisco et al., 2011 |
|  |  | SP50a | 2003 | GU301998 | Francisco et al., 2011 |
|  |  | SP54a | 2003 | GU301999 | Francisco et al., 2011 |
|  |  | SP55a | 2003 | GU302000 | Francisco et al., 2011 |
|  |  | SP57a | 2003 | GU302001 | Francisco et al., 2011 |
|  | 3 | SP41b | 2003 | GU302188 | Francisco et al., 2011 |
|  |  | SP50b | 2003 | GU302189 | Francisco et al., 2011 |
|  |  | SP56a | 2003 | GU302190 | Francisco et al., 2011 |
|  | 4 | SP38b | 2003 | GU302195 | Francisco et al., 2011 |
|  | 5 | SP51a | 2003 | GU302208 | Francisco et al., 2011 |
|  | 6 | SP39b | 2003 | GU302220 | Francisco et al., 2011 |
|  | 7 | SP47a | 2003 | GU302221 | Francisco et al., 2011 |
|  | 8 | SP47b | 2003 | GU302241 | Francisco et al., 2011 |
|  | 9 | SP47b | 2003 | GU983861 | Francisco et al., 2011 |
|  | 10 | CPN64a | 2013 | MH024091 | present work |
|  |  | CPN82a | 2013 | MH024103 | present work |
|  |  | CPN84a | 2013 | MH024106 | present work |
|  |  | CPN85a | 2013 | MH024108 | present work |
|  |  | CPN87a | 2013 | MH024111 | present work |
|  |  | CPN89a | 2013 | MH024114 | present work |
|  |  | CPN90a | 2013 | MH024116 | present work |
|  |  | CPN91a | 2013 | MH024118 | present work |
|  |  | CPN93a | 2013 | MH024120 | present work |
|  |  | CPN96a | 2013 | MH024124 | present work |
|  |  | CPN98a | 2013 | MH024126 | present work |
|  |  | CPN102a | 2013 | MH024134 | present work |
|  |  | CPN104a | 2013 | MH024137 | present work |
|  |  | CPN105a | 2013 | MH024139 | present work |
|  |  | CPN108a | 2013 | MH024143 | present work |
|  |  | CPN109a | 2013 | MH024145 | present work |
|  |  | CPN111a | 2013 | MH024149 | present work |
|  |  | CPN113a | 2013 | MH024151 | present work |
|  |  | CPN115a | 2013 | MH024154 | present work |
|  |  | CPN119a | 2013 | MH024158 | present work |
|  |  | CPN121a | 2013 | MH024160 | present work |
|  |  | CPN124a | 2013 | MH024164 | present work |
|  |  | CPN128a | 2013 | MH024167 | present work |
|  |  | CPN129a | 2013 | MH024169 | present work |
|  |  | CPN130a | 2013 | MH024171 | present work |
|  |  | CPN132a | 2013 | MH024173 | present work |
|  |  | CPN133a | 2013 | MH024175 | present work |
|  |  | CPN136a | 2013 | MH024177 | present work |
|  |  | CPN138a | 2013 | MH024180 | present work |
|  |  | CPN141a | 2013 | MH024184 | present work |
|  |  | CPN147a | 2013 | MH024194 | present work |
|  |  | CPN149a | 2013 | MH024198 | present work |
|  |  | CPN150a | 2013 | MH024200 | present work |
|  |  | CPN154a | 2013 | MH024205 | present work |
|  |  | CPN155a | 2013 | MH024207 | present work |
|  |  | CPN167a | 2013 | MH024217 | present work |
|  |  | CPN185a | 2014 | MH024227 | present work |
|  |  | CPN187a | 2014 | MH024230 | present work |
|  |  | CPN191a | 2014 | MH024235 | present work |
|  |  | CPN193a | 2014 | MH024238 | present work |
|  |  | CPN194a | 2014 | MH024240 | present work |
|  |  | CPN195a | 2014 | MH024242 | present work |
|  |  | CPN198a | 2014 | MH024246 | present work |
|  |  | CPN203a | 2014 | MH024253 | present work |
|  |  | CPN204a | 2014 | MH024255 | present work |
|  |  | CPN206a | 2014 | MH024258 | present work |
|  |  | CPN207a | 2014 | MH024260 | present work |
|  |  | CPN211a | 2014 | MH024265 | present work |
|  |  | CPN213a | 2014 | MH024268 | present work |
|  |  | CPN216a | 2014 | MH024271 | present work |
|  |  | CPN221a | 2014 | MH024279 | present work |
|  |  | CPN222a | 2014 | MH024281 | present work |
|  |  | CPN227a | 2014 | MH024285 | present work |
|  |  | CPN234a | 2014 | MH024293 | present work |
|  |  | CPN236a | 2014 | MH024296 | present work |
|  |  | CPN237a | 2014 | MH024298 | present work |
|  |  | CPN240a | 2014 | MH024302 | present work |
|  |  | CPN242a | 2014 | MH024306 | present work |
|  |  | CPN243a | 2014 | MH024307 | present work |
|  |  | CPN245a | 2014 | MH024311 | present work |
|  |  | CPN247a | 2014 | MH024315 | present work |
|  |  | CPN252a | 2014 | MH024321 | present work |
|  |  | CPN253a | 2014 | MH024323 | present work |
|  |  | CPN257a | 2014 | MH024327 | present work |
|  |  | CPN258a | 2014 | MH024329 | present work |
|  |  | CPN261a | 2014 | MH024331 | present work |
|  |  | CPN262a | 2014 | MH024333 | present work |
|  |  | CPN263a | 2014 | MH024335 | present work |
|  |  | CPN265a | 2014 | MH024339 | present work |
|  |  | CPN267a | 2014 | MH024343 | present work |
|  |  | CPN270a | 2014 | MH024346 | present work |
|  |  | CPN273a | 2014 | MH024350 | present work |
|  |  | CPN274a | 2014 | MH024352 | present work |
|  |  | CPN277a | 2014 | MH024356 | present work |
|  | 11 | CPN64b | 2013 | MH024092 | present work |
|  |  | CPN82b | 2013 | MH024104 | present work |
|  |  | CPN84b | 2013 | MH024107 | present work |
|  |  | CPN85b | 2013 | MH024109 | present work |
|  |  | CPN87b | 2013 | MH024112 | present work |
|  |  | CPN89b | 2013 | MH024115 | present work |
|  |  | CPN90b | 2013 | MH024117 | present work |
|  |  | CPN91b | 2013 | MH024119 | present work |
|  |  | CPN93b | 2013 | MH024121 | present work |
|  |  | CPN96b | 2013 | MH024125 | present work |
|  |  | CPN98b | 2013 | MH024127 | present work |
|  |  | CPN99b | 2013 | MH024129 | present work |
|  |  | CPN100b | 2013 | MH024131 | present work |
|  |  | CPN102b | 2013 | MH024135 | present work |
|  |  | CPN104b | 2013 | MH024138 | present work |
|  |  | CPN105b | 2013 | MH024140 | present work |
|  |  | CPN107b | 2013 | MH024142 | present work |
|  |  | CPN108b | 2013 | MH024144 | present work |
|  |  | CPN109b | 2013 | MH024146 | present work |
|  |  | CPN111b | 2013 | MH024150 | present work |
|  |  | CPN113b | 2013 | MH024152 | present work |
|  |  | CPN115b | 2013 | MH024155 | present work |
|  |  | CPN119b | 2013 | MH024159 | present work |
|  |  | CPN121b | 2013 | MH024161 | present work |
|  |  | CPN124b | 2013 | MH024165 | present work |
|  |  | CPN128b | 2013 | MH024168 | present work |
|  |  | CPN129b | 2013 | MH024170 | present work |
|  |  | CPN130b | 2013 | MH024172 | present work |
|  |  | CPN132b | 2013 | MH024174 | present work |
|  |  | CPN133b | 2013 | MH024176 | present work |
|  |  | CPN136b | 2013 | MH024178 | present work |
|  |  | CPN138b | 2013 | MH024181 | present work |
|  |  | CPN141b | 2013 | MH024185 | present work |
|  |  | CPN145b | 2013 | MH024191 | present work |
|  |  | CPN146b | 2013 | MH024193 | present work |
|  |  | CPN147b | 2013 | MH024195 | present work |
|  |  | CPN148b | 2013 | MH024197 | present work |
|  |  | CPN149b | 2013 | MH024199 | present work |
|  |  | CPN150b | 2013 | MH024201 | present work |
|  |  | CPN154b | 2013 | MH024206 | present work |
|  |  | CPN155b | 2013 | MH024208 | present work |
|  |  | CPN157b | 2013 | MH024210 | present work |
|  |  | CPN161b | 2013 | MH024213 | present work |
|  |  | CPN167b | 2013 | MH024218 | present work |
|  |  | CPN177b | 2014 | MH024222 | present work |
|  |  | CPN183b | 2014 | MH024226 | present work |
|  |  | CPN185b | 2014 | MH024228 | present work |
|  |  | CPN187b | 2014 | MH024231 | present work |
|  |  | CPN188a | 2014 | MH024232 | present work |
|  |  | CPN191b | 2014 | MH024236 | present work |
|  |  | CPN193b | 2014 | MH024239 | present work |
|  |  | CPN194b | 2014 | MH024241 | present work |
|  |  | CPN195b | 2014 | MH024243 | present work |
|  |  | CPN198b | 2014 | MH024247 | present work |
|  |  | CPN200b | 2014 | MH024250 | present work |
|  |  | CPN203b | 2014 | MH024254 | present work |
|  |  | CPN204b | 2014 | MH024256 | present work |
|  |  | CPN206b | 2014 | MH024259 | present work |
|  |  | CPN207b | 2014 | MH024261 | present work |
|  |  | CPN208b | 2014 | MH024263 | present work |
|  |  | CPN211b | 2014 | MH024266 | present work |
|  |  | CPN213b | 2014 | MH024269 | present work |
|  |  | CPN221b | 2014 | MH024280 | present work |
|  |  | CPN222b | 2014 | MH024282 | present work |
|  |  | CPN227b | 2014 | MH024286 | present work |
|  |  | CPN233b | 2014 | MH024292 | present work |
|  |  | CPN234b | 2014 | MH024294 | present work |
|  |  | CPN236b | 2014 | MH024297 | present work |
|  |  | CPN237b | 2014 | MH024299 | present work |
|  |  | CPN240b | 2014 | MH024303 | present work |
|  |  | CPN242b | 2014 | MH024305 | present work |
|  |  | CPN243b | 2014 | MH024308 | present work |
|  |  | CPN247b | 2014 | MH024316 | present work |
|  |  | CPN252b | 2014 | MH024322 | present work |
|  |  | CPN253b | 2014 | MH024324 | present work |
|  |  | CPN257b | 2014 | MH024328 | present work |
|  |  | CPN258b | 2014 | MH024330 | present work |
|  |  | CPN261b | 2014 | MH024332 | present work |
|  |  | CPN262b | 2014 | MH024334 | present work |
|  |  | CPN263b | 2014 | MH024336 | present work |
|  |  | CPN265b | 2014 | MH024340 | present work |
|  |  | CPN267b | 2014 | MH024344 | present work |
|  |  | CPN270b | 2014 | MH024347 | present work |
|  |  | CPN273b | 2014 | MH024351 | present work |
|  |  | CPN274b | 2014 | MH024353 | present work |
|  |  | CPN277b | 2014 | MH024357 | present work |
|  | 12 | CPN68a | 2013 | MH024093 | present work |
|  |  | CPN78a | 2013 | MH024097 | present work |
|  |  | CPN79a | 2013 | MH024099 | present work |
|  | 13 | CPN68b | 2013 | MH024094 | present work |
|  |  | CPN78b | 2013 | MH024098 | present work |
|  |  | CPN79b | 2013 | MH024100 | present work |
|  | 14 | CPN69b | 2013 | MH024096 | present work |
|  |  | CPN80b | 2013 | MH024102 | present work |
|  | 15 | CPN95a | 2013 | MH024122 | present work |
|  | 16 | CPN95b | 2013 | MH024123 | present work |
|  | 17 | CPN99a | 2013 | MH024128 | present work |
|  |  | CPN100a | 2013 | MH024130 | present work |
|  |  | CPN107a | 2013 | MH024141 | present work |
|  |  | CPN145a | 2013 | MH024190 | present work |
|  |  | CPN146a | 2013 | MH024192 | present work |
|  |  | CPN148a | 2013 | MH024196 | present work |
|  |  | CPN157a | 2013 | MH024209 | present work |
|  |  | CPN161a | 2013 | MH024212 | present work |
|  |  | CPN177a | 2014 | MH024221 | present work |
|  |  | CPN183a | 2014 | MH024225 | present work |
|  |  | CPN200a | 2014 | MH024249 | present work |
|  |  | CPN208a | 2014 | MH024262 | present work |
|  |  | CPN233a | 2014 | MH024291 | present work |
|  | 18 | CPN101a | 2013 | MH024132 | present work |
|  | 19 | CPN101b | 2013 | MH024133 | present work |
|  | 20 | CPN110a | 2013 | MH024147 | present work |
|  | 21 | CPN110b | 2013 | MH024148 | present work |
|  | 22 | CPN116a | 2013 | MH024156 | present work |
|  |  | CPN143a | 2013 | MH024187 | present work |
|  |  | CPN168a | 2013 | MH024219 | present work |
|  |  | CPN218a | 2014 | MH024273 | present work |
|  |  | CPN231a | 2014 | MH024289 | present work |
|  |  | CPN244a | 2014 | MH024309 | present work |
|  |  | CPN246a | 2014 | MH024314 | present work |
|  |  | CPN264a | 2014 | MH024337 | present work |
|  |  | CPN266a | 2014 | MH024341 | present work |
|  | 23 | CPN144 | 2013 | MH024189 | present work |
|  | 24 | CPN188b | 2014 | MH024233 | present work |
|  | 25 | CPN196b | 2014 | MH024245 | present work |
|  | 26 | CPN219b | 2014 | MH024276 | present work |
|  | 27 | CPN220a | 2014 | MH024277 | present work |
|  | 28 | CPN220b | 2014 | MH024278 | present work |
|  | 29 | CPN229 | 2014 | MH024287 | present work |
|  | 30 | CPN250a | 2014 | MH024318 | present work |
|  |  |  |  |  |  |
